# Unraveling the Dynamics of Host–Microbiota Indole Metabolism: An Investigation of Indole, Indolin-2-one, Isatin, and 3-Hydroxyindolin-2-one

**DOI:** 10.3390/molecules29050993

**Published:** 2024-02-24

**Authors:** Arnas Kunevičius, Mikas Sadauskas, Julija Raudytė, Rolandas Meškys, Aurelijus Burokas

**Affiliations:** 1 Department of Biological Models, Institute of Biochemistry, Life Sciences Center, Vilnius University, Sauletekio av. 7, LT-10257 Vilnius, Lithuania; 2 Department of Molecular Microbiology and Biotechnology, Institute of Biochemistry, Life Sciences Center, Vilnius University, Sauletekio av. 7, LT-10257 Vilnius, Lithuania; mikas.sadauskas@bchi.vu.lt (M.S.);

**Keywords:** indole, metabolism, indolin-2-one, isatin, 3-hydroxyindolin-2-one

## Abstract

The gut microbiota produces a variety of bioactive molecules that facilitate host–microbiota interaction. Indole and its metabolites are focused as possible biomarkers for various diseases. However, data on indole metabolism and individual metabolites remain limited. Hence, we investigated the metabolism and distribution of indole, indolin-2-one, isatin, and 3-hydroxyindolin-2-one. First, we orally administered a high dose of indole into C57BL/6J mice and measured the concentrations of indole metabolites in the brain, liver, plasma, large and small intestines, and cecum at multiple time points using HPLC/MS. Absorption in 30 min and full metabolization in 6 h were established. Furthermore, indole, indolin-2-one, and 3-hydroxiindolin-2-one, but not isatin, were found in the brain. Second, we confirmed these findings by using stable isotope-carrying indole. Third, we identified 3-hydroxyindolin-2-one as an indole metabolite in vivo by utilizing a 3-hydroxyindolin-2-one-converting enzyme, IifA. Further, we confirmed the ability of orally administered 3-hydroxyindolin-2-one to cross the blood–brain barrier in a dose-dependent manner. Finally, we detected upregulation of the *CYP1A2* and *CYP2A5* genes, confirming the importance of these cytochrome isoforms in indole metabolism in vivo. Overall, our results provide a basic characterization of indole metabolism in the host and highlight 3-hydroxyindolin-2-one as a potentially brain-affecting indole metabolite.

## 1. Introduction

The human gut hosts an extensive variety of microorganisms with almost unlimited metabolic potential. It consists of viruses, bacteria, archaea, fungi, and protozoa, producing approximately 3.3 million unique genes [1,2,3]. These microorganisms contribute to the overall genetic diversity associated with the human population and play an essential role in human health and development [4]. The gut microbiota contributes to a variety of metabolic activities, such as the maintenance of the intestinal barrier, the synthesis of vitamins, the breakdown of otherwise undigestible fibers, and the formation and modulation of the host immune system [5,6]. The complex interactions between the gut microbiota and the host facilitate a deeper insight into the potential molecular targets and mechanisms of action [7]. 

One of the major intercellular signals in the gut microbiota–host interactions is an aromatic amino acid derivative, indole. It is produced from the essential amino acid tryptophan, derived from dietary protein [8], and its metabolism is regulated by the gut microbiota, which affects the gastrointestinal functions of the organism [9]. Approximately 5% of the ingested tryptophan is metabolized by the gut microorganisms, facilitated by the enzyme tryptophanase [10]. More than 85 species of both Gram-positive and Gram-negative bacteria (for example *Clostridium* spp., *Bacteroides* spp., and *Escherichia coli*) contain the *tnaA* gene encoding tryptophanase. Furthermore, even relatively small amounts of indole have an important interbacterial and host physiological effect [8,11]. As a signaling molecule of the bacterial population, it regulates cell growth [12], the formation of biofilm [13], resistance to acid [14], virulence [15], and stable plasmid expression [16]. Additionally, a lot of attention has been brought to the gut microbiota metabolizing indole into a plethora of bioactive metabolites, such as indole-3-ethanol, indole-3-propionic acid, indole-3-lactic acid, indole-3-acetic acid, skatole, indole-3-carboxaldehyde, and indole-3-acrylic acid [10,17,18,19,20]. However, the knowledge about the indole metabolites produced by the host is limited. 

**Figure 1:**
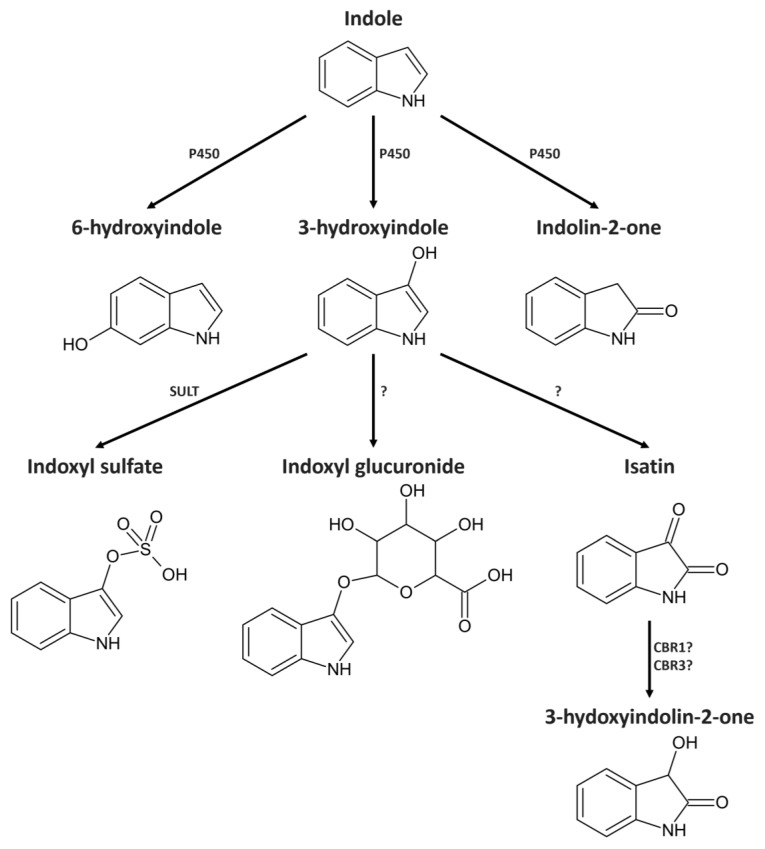
Possible metabolic pathway of indole metabolism in vivo. Adapted from [22,23,31,32]. P450—cytochrome 450, SULT—sulfotransferase, CBR1—carbonyl reductase type 1. CBR3—carbonyl reductase type 3.

Extensive changes in the gut microbiota composition can lead to an increase in indole-producing bacteria or an impaired intestinal barrier. This creates conditions that increase the concentration of indole inside the host [21]. After absorption, indole is usually further metabolized in the liver by enzymes of the cytochrome P450 superfamily (Figure 1). In vitro data indicate that CYP2A6, CYP2C19, and CYP2E1 have the highest activity in the metabolization of indole [22]. The most important bioactive metabolites in this process are indolin-2-one (IUPAC 1,3-dihydro-2*H*-indol-2-one), isatin (IUPAC 3-hydroxy-2*H*-indol-2-one), and 3-hydroxyindolin-2-one (IUPAC 3-hydroxy-1,3-dihydro-2*H*-indol-2-one). Furthermore, experiments with hepatic microsomes suggest that the majority of indole is hydroxylated by CYP2E1 to generate an intermediate metabolite 3-hydroxyindole and finally conjugated by sulfotransferase to produce indoxyl sulfate, which is effectively excreted with urine [23].

Limited data are available on the biological activity of indole metabolites. Indolin-2-one and isatin have been found to cross the blood–brain barrier and have an inhibitory effect on the CNS and behavior [24]. Low doses (10 mg/kg) of indolin-2-one in the blood cause reduced blood pressure and mild sedation, while high doses (100 mg/kg) can cause coma and death [25]. Isatin is a potent inhibitor of monoamine oxidase B, an enzyme responsible for the synthesis of the neurotransmitter GABA [26,27]. However, most of the knowledge on the molecular targets of isatin comes from in vitro studies. It most prominently inhibits the atrial natriuretic peptide signaling system, which regulates osmotic pressure in the kidneys and hypertrophic function in the heart [28]. This indicates the role of isatin in kidney malfunction-induced heart hypertrophy. 3-Hydroxyindolin-2-one, a secondary metabolite of isatin, is even more obscure. It was discovered in *E. coli* expressing P450 enzymes after indole exposure and occurs naturally in plants and fungi [22,29,30]. It is also an intermediate metabolite in the bacterial indole metabolism [20]. There are no in vivo data on the ability of 3-hydroxyindolin-2-one to cross the blood–brain barrier or its possible physiological effects on the nervous system of animals. 

Due to the increasing discussion on the use of indole metabolites as biomarkers for various diseases [33,34,35], there is a need for a more comprehensive characterization of indole metabolism. Especially, host-derived indole metabolites have been implicated in many disorders without understanding the underlying metabolism and pharmacokinetics in vivo [19]. Investigating the permeability of indole metabolites through the intestinal and blood–brain barriers increases the understanding of which compounds could have a systemic effect and contribute to the development of different microbiota-associated disorders. This would act as a stepping stone for improving biomarker detection timelines and strategies. Furthermore, it could reveal previously unknown compounds that could be utilized as clinical targets or important research molecules. Hence, we investigated the distribution and temporal profile of three indole metabolites (indolin-2-one, isatin, and 3-hydroxyindolin-2-one) in vivo. First, we orally administered a high dose of indole into C57BL/6J mice and measured the concentrations of indole metabolites in the brain, liver, plasma, large and small intestines, and cecum at multiple time points (30 min, 1 h, 2 h, 4 h, and 6 h) using HPLC/MS. Second, we confirmed these findings using stable isotope-carrying indole. Third, we confirmed 3-hydroxyindolin-2-one as an indole metabolite in vivo. Finally, we investigated changes in gene expression of enzymes involved in indole metabolism. Overall, we established that 3-hydroxyindolin-2-one, similar to indolin-2-one, crosses the blood–brain barrier and can affect the CNS. However, the metabolites investigated in this study are metabolized within 6 h, and their use as biomarkers remains difficult. 

## 2. Results

### 2.1. Indole Is Quickly and Efficiently Metabolized In Vivo 

To clarify the properties of indole metabolism and metabolites in vivo, the concentrations of indole and its metabolites (indolin-2-one and isatin) were measured in different organs (small intestine, cecum, large intestine, liver, plasma, and brain) at different time intervals (30 min, 1 h, 2 h, 4 h, and 6 h). The extraction efficiency, measured by the amount of recovered *N*-methylindolin-2-one, used as an internal standard, was 50–70% for most samples (Tables S1–S6). Only a few samples had extraction efficiency below 30%, and these samples were also included in the data analysis.

**Figure 2:**
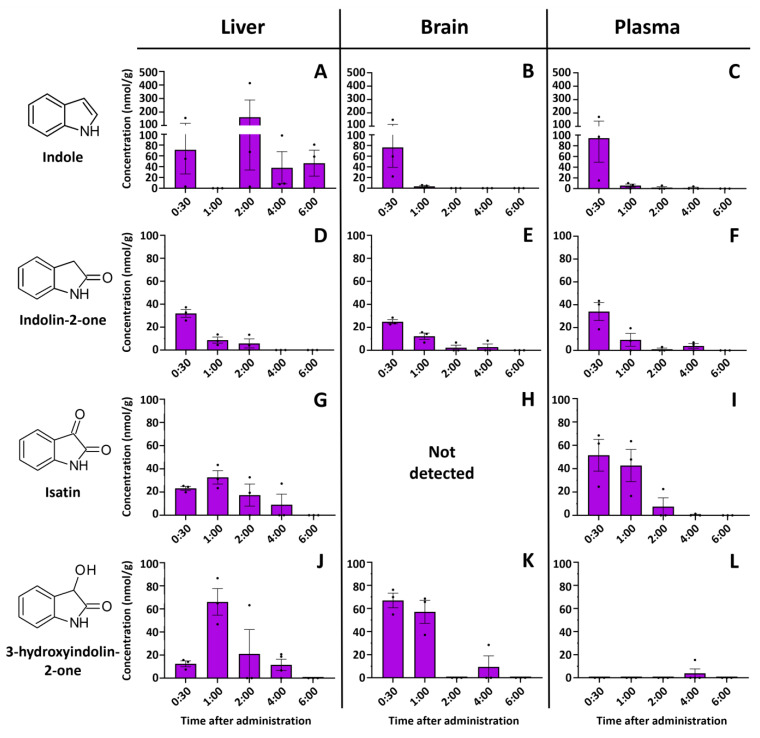
Time-resolved concentrations of (**A**)—indole in the liver, (**B**)—indole in brain, (**C**)—indole in plasma, (**D**)—indolin-2-one in liver, (**E**)—indolin-2-one in brain, (**F**)—indolin-2-one in plasma, (**G**)—isatin in liver, (**H**)—isatin was not detected in the brain, (**I**)—isatin in plasma, (**J**)—3-hydroxyindolin-2-one in liver, (**K**)—3-hydroxyindolin-2-one in brain, (**L**)—3-hydroxyindolin-2-one in plasma after oral administration of indole. Control samples did not contain any of the compounds investigated. Concentrations are shown in nmol/per gram of tissue. Data represented as means ± SEM. N = 3 for all time points. Dots represent individual sample values.

Absorption of indole, even when dissolved in a lipid solvent, is rapid, and a significant concentration is found in all major organs 30 min after ingestion (Figure 2). The highest indole concentration was found in the liver 2 h after ingestion, reaching 414.15 nmol/g. Indole was also detected in the brain and plasma, but only at the beginning of detoxification (30 min after injection). Meanwhile, the indole metabolites indolin-2-one and isatin were detected over longer periods (up to 6 h after injection) but at lower concentrations than the indole concentration. Indole or any investigated metabolites were not detected in the control samples. For example, the concentrations of indolin-2-one and isatin peaked after 30 min of injection and then gradually decreased in the liver, plasma, and brain. Even at this supraphysiological concentration (350 mg/kg), indole is metabolized within 6 h and can only be detected in the intestines afterward. 

**Figure 3:**
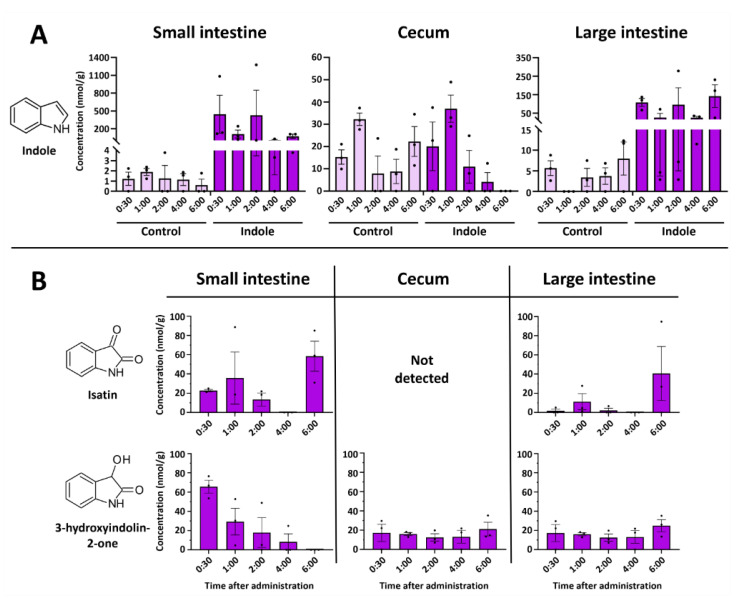
Time-resolved concentrations of indole and its metabolites in the intestines after oral administration of indole. (**A**)—Concentration of indole in the intestinal system. Control samples in intestinal samples contained small amounts of indole at all time points. (**B**)—Concentration of isatin and 3-hydroxyindolin-2-one in the intestines. All control samples did not contain any indole metabolites. Data represented as means ± SEM. N = 3 for all time points.

Major fluctuations in the concentrations of indole and its metabolites were observed in the intestinal tract. Indole showed an expected decrease with time, while isatin concertation peaked at 6 h post-injection (Figure 3A). Surprisingly, indolin-2-one was the only analyzed metabolite not detected in the intestines at any time point, while isatin was not detected in the cecum. On the other hand, a relatively high amount of isatin was found in the small intestine 6 h after indole administration (Figure 3B). Equivalent amounts of indole were detected in the cecum of the indole-administered and control animals. Indole concentration in the cecum was stable across all time points.

Interestingly, we also observed another indole metabolite in the organ extracts—3-hydroxyindolin-2-one (see Section 2.3 for the identification). This compound was detected in all organs tested (Figure 2 and Figure 3B), whereas no 3-hydroxyindolin-2-one was found in any of the organs of the control animals. The highest concentrations were observed in the liver (Figure 2J), small intestine (Figure 3A), and brain (Figure 2) 1 h after administration and generally followed the concentration-decreasing pattern of other indole metabolites. 3-Hydroxyindolin-2-one was detected in all tissues analyzed, with only minimal levels detected in plasma (Figure 2L). 

### 2.2. Indole from the Digestive Tract Is Metabolized by the Host and Produces Multiple Bioactive Metabolites

**Figure 4:**
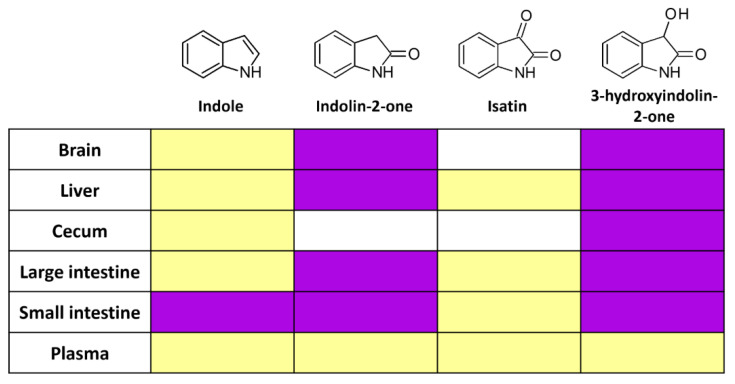
Host utilizes isotope-carrying indole and produces several bioactive metabolites. Yellow—metabolite was detected, but no mass spectra were obtained due to low concentration. Magenta—13C isotope-carrying metabolite was detected, but white—below the detection limit. N = 1.

Since indole is produced endogenously by the gut microbiota from dietary compounds, it is possible that the metabolites identified in the organ extracts were produced from endogenous indole or other precursors rather than the exogenously supplied indole. To clarify the nature of the detected metabolites, we gavaged the mice with the equivalent doses of stable isotope-carrying indole (^13^C at C2 position) and analyzed the organ extracts with the HPLC/MS. We detected heavier isotopes of indolin-2-one and 3-hydroxyindolin-2-one in the brain, liver, cecum, small intestine, and colon 2 h after administration (Figure 4, Figures S1–S15). This confirmed the previous experiment and provided direct evidence that indole in the gut is a substrate for several metabolites that spread throughout the body and even to the brain. 

### 2.3. Identification of 3-Hydroxyindolin-2-one as Indole Metabolite 

**Figure 5:**
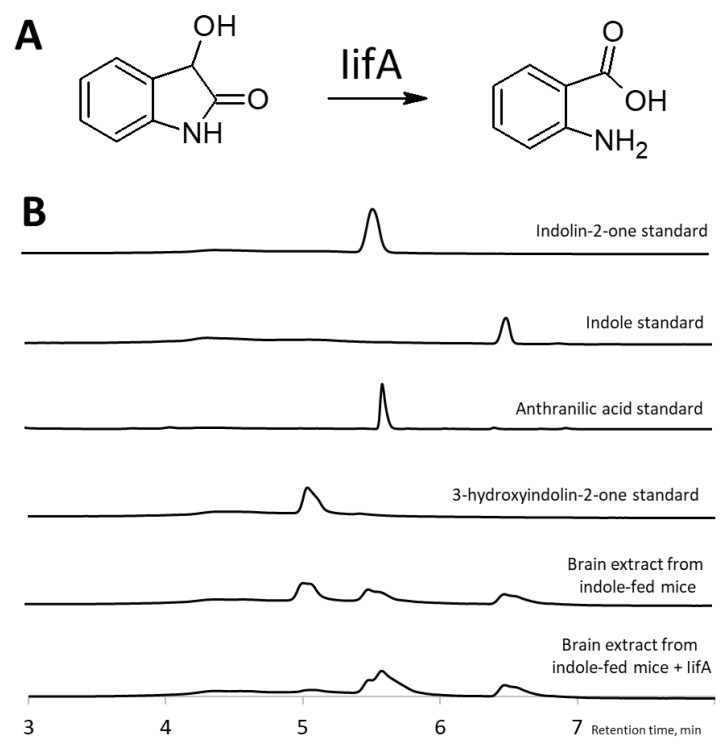
Identification of 3-hydroxyindolin-2-one as indole metabolite. (**A**)—IifA catalyzes the conversion of 3-hydroxyindolin-2-one into anthranilic acid. (**B**)—HPLC chromatograms showing that IifA converts indole metabolite with a retention time of 5.0 min (corresponding to the 3-hydroxyindolin-2-one standard) to a product with a retention time of 5.6 min (corresponding to the anthranilic acid standard).

To confirm the structure of a compound with a retention time of 5.0 min in the organ extracts of indole-fed mice, we performed a reaction with an IifA enzyme [20]. This putative cofactor-independent oxygenase is involved in the bacterial assimilation of indole and catalyzes the conversion of 3-hydroxyindolin-2-one into anthranilic acid. When treated with IifA, the brain extracts of indole-fed mice showed a diminished peak at 5.0 min (corresponding to the 3-hydroxyindolin-2-one standard) and the formation of a new peak at 5.6 min (corresponding to the anthranilic acid standard) (Figure 5). This new compound possessed an absorbance peak at 330 nm and a molecular mass of 138 [M + H]^+^, characteristic of anthranilic acid. Based on the result that IifA converted the compound into anthranilic acid at 5.0 min, it was concluded that this compound was 3-hydroxyindolin-2-one.

### 2.4. Intestinal 3-Hydroxyindolin-2-one Can Cross the Blood–Brain Barrier

**Figure 6:**
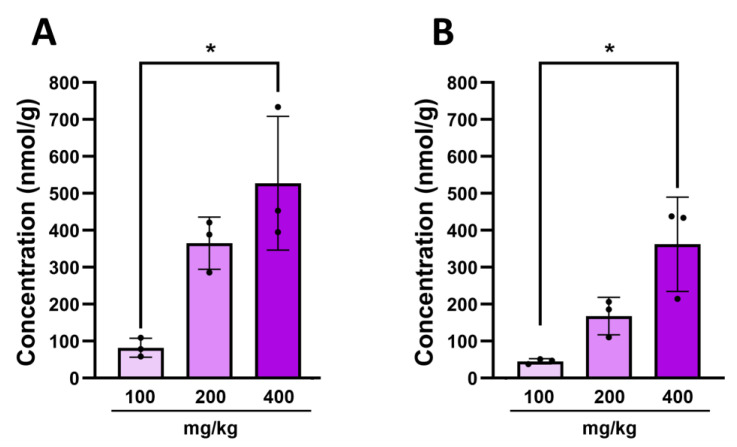
A dose-dependent increase in concentration of 3-hydroxyindolin-2-one after oral administration. (**A**)—concentration in plasma (**B**)—concentration in brain. Data represented as means ± SEM. N = 3 for all doses. The Kruskal–Wallis H test was used to detect significant differences in 3-hydroxyindolin-2-one treated animals (3 animals per group, * *p* ≤ 0.05).

We have detected the presence of 3-hydroxyindolin-2-one in the brain after administration of indole, but there was still no direct evidence that it crosses the blood–brain barrier. Hence, we administered various doses of 3-hydroxyindolin-2-one to healthy adult animals and observed a significant dose-dependent increase in the metabolite concentration in the plasma (Figure 6A) and brain (Figure 6B) of these animals 30 min after ingestion. 3-Hydoxyindolin-2-one was not detected in any of the control animals. This highlights the ability of 3-hydroxyindolin-2-one to cross both the intestinal and the blood–brain barrier and to have a neuroinhibitory effect on neuronal activity and behavior. 

### 2.5. Gene Expression Changes Caused by Indole Administration

**Figure 7:**
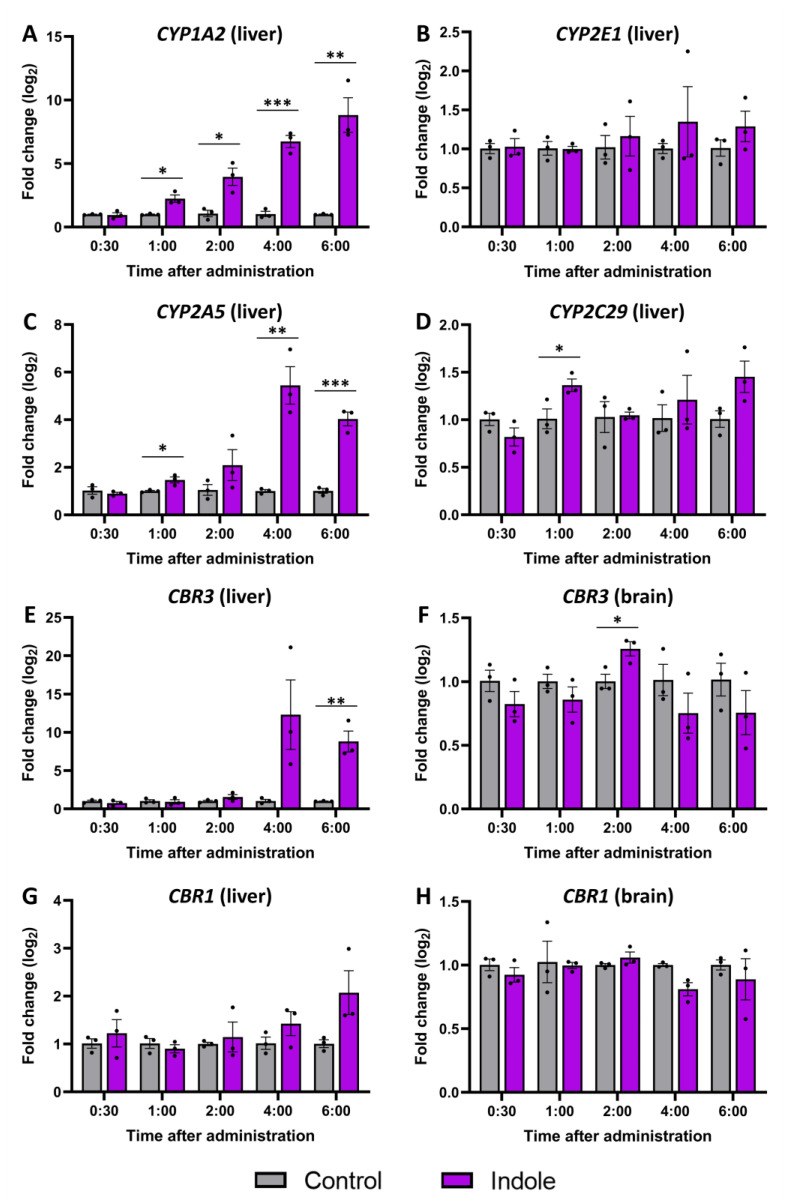
Gene expression changes caused by indole administration. Expression of P450 family enzymes-encoding genes: (**A**)—*CYP1A2*, (**B**)—*CYP2E1*, (**C**)—*CYP2A5*, (**D**)—*CYP2C29*. Expression of *CBR3* in (**E**)—liver, (**F**)—plasma. *CBR1* in (**G**)—liver, (**H**)—plasma. Vehicle control in gray and indole-administered in magenta. The normalized fold change ± SEM was calculated using the delta-delta Ct method with *GAPDH* (Glyceraldehyde 3-phosphate dehydrogenase) as a reference gene. The Student’s *t*-test was used to detect significant differences in control and indole-treated animals at each time point (3 animals per group, * *p* ≤ 0.05, ** *p* ≤ 0.01, *** *p* ≤ 0.001).

A gene expression analysis was performed to determine which isoforms of liver cytochrome P450 enzymes are responsible for indole metabolism in vivo and establish an expression profile (Figure 7). The *CYP1A2* isoform (Figure 7A) showed the highest overall expression, and a significant increase was observed 1 h after indole administration, increasing with time. *CYP2A5* (Figure 7C) showcased a similar expression profile with a drastic increase 4 and 6 h after administration. Meanwhile, the expression of *CYP2E1* (Figure 7B) and *CYP2C29* (Figure 7D) was mainly unchanged. Also, we examined the expression of *CBR1* and *CBR3*, the two isoforms encoding carbonyl reductase, which facilitates the conversion of isatin to 3-hydroxyindolin-2-one. Analogous to *CYP2A5*, the expression of *CBR3* in the liver (Figure 7E) sharply increased 4 and 6 h (12 and 8 times, respectively) after indole administration. However, the expression level of *CBR1* was not altered in the liver after the administration of indole. To check if 3-hydroxyindolin-2-one can be produced inside the CNS, the expression of carbonyl reductase types 1 and 3 (*CBR1* and *CBR3*) was investigated in the brain (Figure 7F,H). We detected a slight increase in *CBR3* expression 2 h after indole administration. *CBR1* expression remained unaffected by indole administration at any time iepoint in both the liver and brain. Overall, gene expression data hint that the reduction of isatin and production of 3-hydroxyindolin-2-one is located in the liver and possibly mediated by CBR3.

## 3. Discussion

Indole is produced from dietary tryptophan by the gut microbiota and enters the body through the intestinal epithelium [19]. Indole is metabolized in the liver, but the complete metabolic pathway and specific intermediate metabolites are still debated [22,23,31,35]. Therefore, in this study, we investigated the metabolism of the aromatic heterocyclic compound indole in vivo. We showcased the fast absorption of indole, peaking the plasma concentration at 30 min after oral administration. Furthermore, the majority of the indole was metabolized within 6 h after administration. This indicates a rapid metabolism and turnover rate of indole and argues against using indole or host-produced indole metabolites as potential biomarkers.

Data on the physiological effects of different host-produced indole metabolites remain contradictory and limited. However, elevated concentrations of most indole metabolites, e.g., indoxyl sulfate, indolin-2-one, and isatin, have a toxic effect on various human cells and tissues [26,31]. Furthermore, previous work indicated an inhibitory effect of indolin-2-one and isatin on the CNS [24,25,36]. On the other hand, isatin regulates anti-inflammatory signaling pathways and has an anti-carcinogenic effect [37]. The effect of 3-hydroyindolin-2-one is entirely unknown. Hence, indolin-2-one, isatin, and 3-hydroyindolin-2-one were selected for this study. Interestingly, our data indicate indolin-2-one, but not isatin, in the brain after indole administration. The absence of isatin could be explained either by the conversion of isatin into 3-hydroxyindolin-2-one in the brain by carbonyl reductase type 3 [38] or by the inability of isatin to cross the bloodbrain barrier. However, this is unlikely due to its relatively small size and similar molecular structure. Jaglin and colleagues reported up to 5 nmol/g of isatin in the brain after systemic administration [24]. The absence of isatin in the brain could, therefore, be due to the lack of sensitivity of our detection system.

Besides the brain, the differential distribution of indole metabolites was detected in the intestines, which are the center point of indole production under physiological conditions due to the metabolic activity of the gut microbiota. Indolin-2-one was not detected in the small intestine, cecum, or large intestine, whereas isatin was detected in the small and large intestines but not in the cecum. This shows a different distribution of the indole metabolites. However, the role of the gut microbiota in the production of indolin-2-one and isatin is mainly unknown. Zhang and colleagues reported that indolin-2-one and isatin were produced by recombinant *E. coli* [39]. However, the gut microbiota is unlikely to synthesize physiologically relevant doses of indole metabolites for the host. This is supported by the fact that the intestines were the only organs where control samples contained low levels of indole, up to 15 nmol/g in the intestines and up to 40 nmol/g in the cecum. However, indolin-2-one, isatin, or 3-hydroxyindolin-2-one were not detected in the control samples. Overall, the number of different metabolites was similar in the liver and intestines.

To gain a deeper understanding of indole metabolism, we investigated the changes in gene expression in the liver and the brain after indole administration. The P450 cytochrome isoforms were selected based on previous in vitro work [22,23]. *CYP1A2* and *CYP2A5* showed the most significant upregulation in gene expression indicating their importance in indole metabolism. Surprisingly, we did not observe an increase in gene expression of *CYP2E1* and *CYP2C29*, two isoforms conventionally linked to indole metabolism [17,23,40]. Furthermore, increased expression of *CBR3* in the liver after indole administration aligns with earlier in vivo findings [32], reinforcing the potential significance of CBR3 in the production of 3-hydroxyindolin-2-one and the broader metabolism of indole. Conversely, the expression of *CBR1* was not altered by indole administration in either the liver or brain, despite the reported higher enzymatic activity and higher level of expression [32,38,40]. Our results indicate that *CBR3,* rather than *CBR1,* plays a role in the reduction of isatin and synthesis of 3-hydroxyindolin-2-one in the liver; however, it is likely that other unidentified enzymes contribute to this reaction.

There are very little data on 3-hydroxyindolin-2-one in animals [36], and there are no data on whether it crosses the blood–brain barrier or can be detected inside the CNS. To our knowledge, 3-hydroxyindolin-2-one has never been directly shown to be metabolized from indole in vivo, but only in *E. coli* expressing cytochrome P450 enzymes [22]. Although we detected only a minute amount of 3-hydroxyindolin-2-one in the plasma 4 h after administration, the metabolite was detected in the brain as early as 30 min after administration and for 4 h. We confirmed that 3-hydroxyindolin-2-one and indolin-2-one are metabolites of the administered indole by using isotope-carrying indole. We detected a heavier isotope of 3-hydroxyindolin-2-one in the brain, liver, cecum, and small intestine. The presence of 3-hydroxyindolin-2-one in the brain was further confirmed utilizing the IifA enzymatic reaction. IifA catalyzes oxygenation of 3-hydroxyindolin-2-one into anthranilic acid. Brain extracts from indole-administered animals treated with IifA yielded chromatograms typical of anthranilic acid, thus confirming the presence of 3-hydroxyindolin-2-one in the brain. Finally, we confirmed the ability of 3-hydroxyindolin-2-one to cross both the intestinal and blood–brain barriers. After oral administration, it was detected in the brain in a dose-dependent manner. All data suggest that gut-derived indole is a substrate for a novel metabolite—3-hydroxyindolin-2-one—that crosses the blood–brain barrier.

The physiological effect of 3-hydroxyindolin-2-one in animals is largely unknown. In vitro data hint at an anti-thrombotic effect through modulation of the platelet function [41]. Usami and colleagues reported that 3-hydroxyindolin-2-one has a lower inhibitory effect on monoamine oxidase and acetylcholinesterase compared to isatin [32]. In vivo studies report the inhibitory effect of indolin-2-one and isatin [24] on the CNS. Thus, it is most probable that 3-hydroxyindolin-2-one has a similar effect. However, further studies on neuronal activity and animal levels of activity and anxiety are needed to confirm these claims.

## 4. Materials and Methods

### 4.1. Materials

Materials and chemicals were purchased from the following suppliers: Sigma-Aldrich, St. Louis, MO, USA (indole, isatin, indolin-2-one, *N*-methylindolin-2-one, and methanol), Eurisotop, Bristol, UK (indole-^13^C), Honeywell, Charlotte, NC, USA (ethyl acetate), Animalab, Kraków, Poland (feeding tubes).

### 4.2. Animals

**Figure 8:**
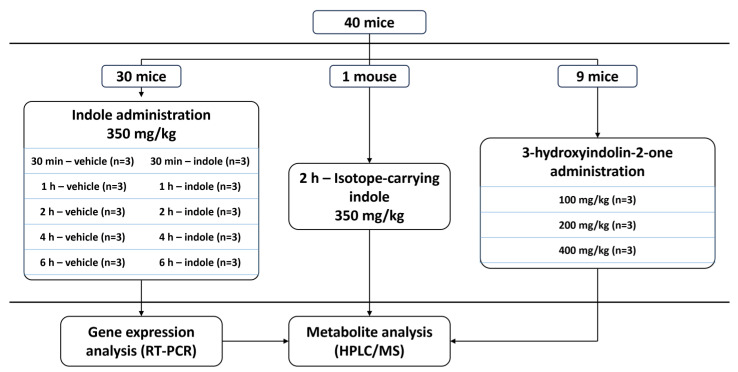
Experimental design scheme.

All animal testing was performed with the European Communities Council Directive 2010/63/E.U. guidelines and approved by the Lithuania State Food and Veterinary Service, Animal Ethics Experimentation Committee (project G2-244). C57BL/6JRj mouse line animals (Janvier Labs, Le Genest-Saint-Isle, France) were used for all procedures. Before the experiment, animals were housed in groups under a 12 h light–dark cycle with a temperature of 21 ± 1 °C and humidity of 55 ± 10%. The experiment was conducted in 3 major phases (Figure 8). First, 5 time points were chosen, and 6 animals per time point (3 treated with 200 μL of oil-based indole solution and 3 control animals that received vehicle) were used to establish a temporal profile of indole metabolism using HPLC/MS. Liver and brain tissues from the same animals were then used to determine gene expression changes caused by increased indole concentration in the digestive system. Second, the temporal metabolism data were validated by a qualitative step using one mouse treated with isotope-carrying indole (^13^C at C2 position). Finally, nine mice were orally administered with 3-hydroxyindolin-2-one to showcase its ability to cross the blood–brain barrier.

### 4.3. Administration of Indole and 3-Hydroxyindolin-2-one

Indole and 3-hydroxyindolin-2-one were dissolved in sterile vegetable oil. Animals were starved for 1 h before the administration of the compounds. Indole was administered by gavage at the dose of 350 mg/kg body weight, gavaged volume—200 µL. The animals then were housed in single cages and culled at different time points (30 min, 1 h, 2 h, 4 h, and 6 h). The organs were immediately extracted on ice and stored at –80 °C until further analysis. The inside of the intestines was cleaned before freezing. 3-Hydroxyindolin-2-one was administered by gavage at three doses (100 mg/kg, 200 mg/kg, and 400 mg/kg). The animals were culled 30 min after the administration. The organs were immediately removed on ice and stored at –80 °C until further analysis.

### 4.4. Liquid–Liquid Extraction of Indole and Its Metabolites 

Indole and its metabolites were extracted by adapting a mixed protocol from the literature [24]. Organ samples were thawed, weighed, and homogenized (3 cycles ×30 s, using Benchmark the BeadBug 6 homogenizer, Sayreville, NJ, USA) in 1 mL of deionized water containing different concentrations of 1-methylindolin-2-one as an internal standard. Plasma samples were not homogenized and were subjected to liquid–liquid extraction directly. Organ homogenates were centrifuged at 16,000× *g* for 10 min at 4 °C. The supernatants were extracted three times with equal amounts of ethyl acetate. All three extracts were pooled and evaporated under reduced pressure (AES2000 SpeedVAC, Savant, Thermo Fisher Scientific, Waltham, MA, USA). The dried material was dissolved in 100 μL 50% methanol by vigorous shaking and centrifugation at 16,000× *g* for 5 min. The supernatant was subjected to HPLC/MS analysis. 

To assess the extraction efficiency, all samples were spiked with 1-methylindolin-2-one as an internal standard before the extraction. The extraction efficiency was calculated as follows: (A_extracted_/A_added_) × 100%, where A_extracted_—the amount of 1-methylindolin-2-one extracted, added—the amount of 1-methylindolin-2-one added to the sample before extraction.

### 4.5. Quantification of Indole Metabolites by HPLC/MS 

Organ extracts were analyzed by HPLC/MS as described in [20]. The concentrations of indole, *N*-methylindolin-2-one, indolin-2-one, and 3-hydroxyindolin-2-one were determined using standard sample concentrations of these compounds and integrating the peak area from the HPLC chromatograms at 254 nm. HPLC chromatograms at 417 nm were used to calculate isatin concentrations. Limits of detection (LOD) for HPLC determination of the metabolite concentrations were determined by using different concentrations of standard solutions and following the same extraction protocol as described above. The LOD values were as follows: indole—0.5 nmol/g, indolin-2-one—2 nmol/g, 3-hydroxyindolin-2-one—1 nmol/g, isatin—1 nmol/g.

### 4.6. Gene Expression Analysis by Real-Time PCR (RT-PGR)

The total RNA was isolated from the mice’s brain medulla and liver tissues using the TRIzol™ Plus RNA Purification Kit according to the manufacturer’s protocol (Thermo Fisher Scientific, Vilnius, Lithuania). To remove gDNA, samples were treated with dsDNase reagent (Thermo Fisher Scientific, Lithuania). cDNA was synthesized using a High-Capacity cDNA Reverse Transcription kit (Thermo Fisher Scientific, Lithuania) according to the manufacturer’s instructions. The expression of *CYP1A2*, *CYP2E1*, *CYP2A5*, *CYP2C29*, *CBR1, CBR3*, and *GAPDH* was analyzed by the QuantStudio™ 3 Real-Time PCR System (Thermo Fisher Scientific, Lithuania), using SYBR™ Green PCR Master Mix (Thermo Fisher Scientific, Lithuania). The following primer sequences were used: Cyp2c29_FW (5′-GCTCTCCTACTCCTGCTGAAGT-3′), Cyp2c29_RV (5′-ATGTGGCTCCTGTCTTGCATGC-3′), Cyp2a5_FW (5′-GGAAGACGAACGGTGCTTTT-3′), Cyp2a5_RV (5′-TTCCCAGCATCATTCGAAGC-3′), Cbr3_FW (5′-GGGCATCGCCTTTAGAATGGA-3′), Cbr3_RV (5′-GGTCCACCTCGGTAAGTGTG-3′), Cyp1a2_FW (5′-GACATGGCCTAACGTGCAGA-3′), Cyp1a2_RV (5′-GTGTCCCTCGTTGTGCTGTG-3′), Cyp2e1_FW (5′-GCTCAAAAAGACCAAAGGCCAG-3′), Cyp2e1_RV (5′-GACTTTTCTGTGGCTTCCAGG-3′), GAPDH_FW (5′-GGCATTGTGGAAGGGCTCAT-3′), GAPDH_RV (5′-AGATCCACGACGGACACATT-3′), Cbr1_FW (5′-CCTCTAATAAAACCCCAAGGCAG-3′), Cbr1_RV (5′-GCTCCTCCTCTGTGATGGTC-3′). All samples were run in duplicates. Ct values were normalized to *GAPDH* using the delta–delta Ct method.

### 4.7. Statistical Analysis

All grouped analysis data were presented as mean ± SEM. RT-PCR data normality was established using the Shapiro–Wilk normality test, and the parametric unpaired Student’s *t*-test was used to determine statistically significant differences (* *p* ≤ 0.05 ** *p* ≤ 0.01, *** *p* ≤ 0.001). The Kruskal–Wallis H test was used to detect significant differences in the administration of 3-hydroxyindolin-2-one (* *p* ≤ 0.05 ** *p* ≤ 0.01, *** *p* ≤ 0.001).

## Data Availability

Data are contained within the article.

## Supplementary Materials

The following supporting information can be downloaded at: https://www.mdpi.com/article/10.3390/molecules29050993/s1, Figure S1: HPLC profile, mass spectra (in positive ionization mode) and UV absorbance spectrum of indole standard; Figure S2: HPLC profile, mass spectra (in positive ionization mode) and UV absorbance spectrum of indolin-2-one standard; Figure S3: HPLC profile, mass spectra (in positive ionization mode) and UV absorbance spectrum of isatin standard; Figure S4: HPLC profile, mass spectra (in positive ionization mode) and UV absorbance spectrum of 1-methylindolin-2-one standard; Figure S5: HPLC profile, mass spectra (in positive ionization mode) and UV absorbance spectrum of 3-hydroxyindolin-2-one standard; Figure S6: HPLC profile, mass spectra (in positive ionization mode) and UV absorbance spectrum of indole, indolin-2-one, isatin, and 3-hydroxyindolin-2-one in small intestine; Figure S7: HPLC profile, mass spectra (in positive ionization mode) and UV absorbance spectrum of indole, indolin-2-one, isatin, and 3-hydroxyindolin-2-one in small intestine after 13C isotope-carrying indole administration; Figure S8: HPLC profile, mass spectra (in positive ionization mode) and UV absorbance spectrum of indole, indolin-2-one, isatin, and 3-hydroxyindolin-2-one in cecum; Figure S9: HPLC profile, mass spectra (in positive ionization mode) and UV absorbance spectrum of indole, indolin-2-one, isatin, and 3-hydroxyindolin-2-one in cecum after 13C isotope-carrying indole administration; Figure S10: HPLC profile, mass spectra (in positive ionization mode) and UV absorbance spectrum of indole, indolin-2-one, isatin, and 3-hydroxyindolin-2-one in large intestine; Figure S11: HPLC profile, mass spectra (in positive ionization mode) and UV absorbance spectrum of indole, indolin-2-one, isatin, and 3-hydroxyindolin-2-one in large intestine after 13C isotope-carrying indole administration; Figure S12: HPLC profile, mass spectra (in positive ionization mode) and UV absorbance spectrum of indole, indolin-2-one, isatin, and 3-hydroxyindolin-2-one in liver; Figure S13: HPLC profile, mass spectra (in positive ionization mode) and UV absorbance spectrum of indole, indolin-2-one, isatin, and 3-hydroxyindolin-2-one in liver after 13C isotope-carrying indole administration; Figure S14: HPLC profile, mass spectra (in positive ionization mode) and UV absorbance spectrum of indole, indolin-2-one, isatin, and 3-hydroxyindolin-2-one in brain; Figure S15: HPLC profile, mass spectra (in positive ionization mode) and UV absorbance spectrum of indole, indolin-2-one, isatin, and 3-hydroxyindolin-2-one in brain after 13C isotope-carrying indole administration; Table S1: Concentrations of indole metabolites in liver extracts; Table S2: Concentrations of indole metabolites in brain extracts; Table S3: Concentrations of indole metabolites in plasma; Table S4: Concentrations of indole metabolites in small intestine (SI) extracts; Table S5: Concentrations of indole metabolites in cecum extracts; Table S6: Concentrations of indole metabolites in large intestine (LI) extracts.
