# Supplementary material for: Unraveling the Dynamics of Host–Microbiota Indole Metabolism: An Investigation of Indole, Indolin-2-one, Isatin, and 3-Hydroxyindolin-2-one
Source: Molecules. 2024 Feb 24;29(5):993. doi: 10.3390/molecules29050993 (PMC10934121; doi:10.3390/molecules29050993)
Supplement: Supplementary file 1 [file molecules-29-00993-s001.zip › molecules-2814041-supplementary.pdf]

Unraveling the Dynamics of Host-Microbiota Indole Metabolism: An Investigation of Indole, Indolin-2-one, Isatin, and 3-Hydroxyindolin-2-one

Supplementary material

Arnas Kunevičius[1]*, Mikas Sadauskas[2], Julija Raudytė[1], Rolandas Meškys[2], Aurelijus Burokas[1]*

[1] Department of Biological Models, Institute of Biochemistry, Life Sciences Center, Vilnius University, Vilnius, Lithuania
[2] Department of Molecular Microbiology and Biotechnology, Institute of Biochemistry, Life Sciences Center, Vilnius University, Vilnius, Lithuania
* Correspondence: AK: arnas.kunevicius@gmc.vu.lt,AB: aurelijus.burokas@gmc.vu.lt

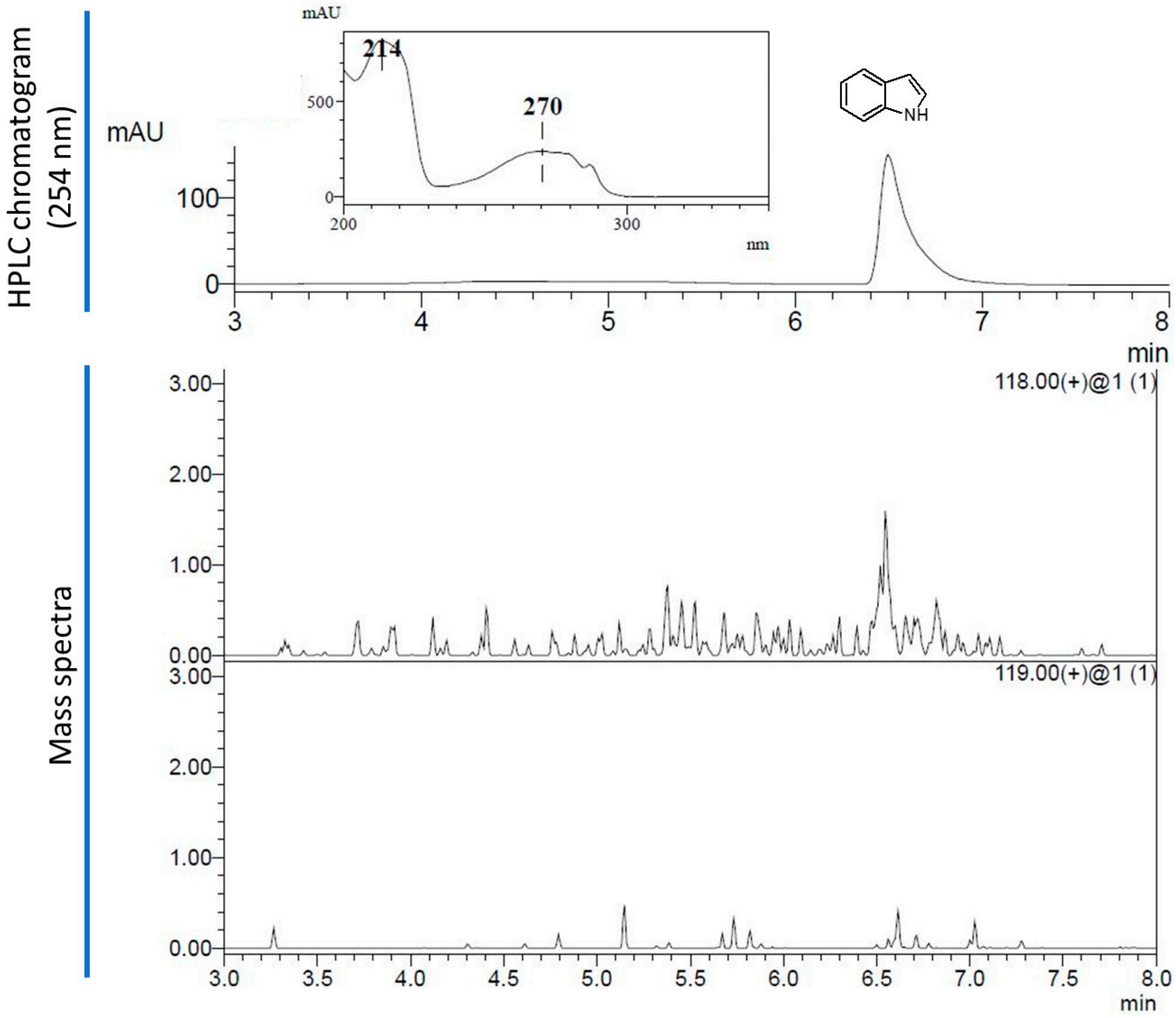

Figure S1. HPLC profile, mass spectra (in positive ionization mode) and UV absorbance spectrum of indole standard.

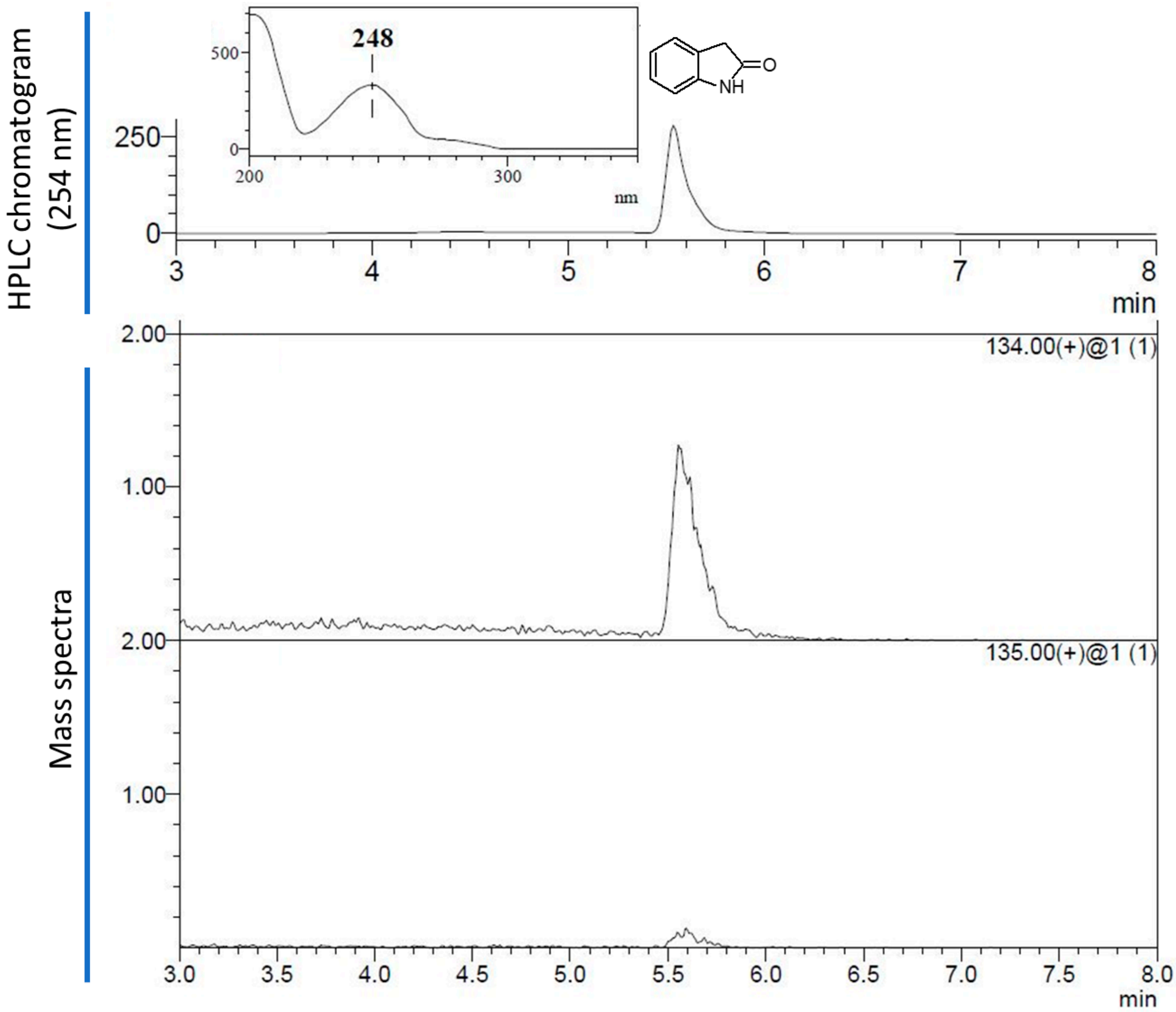

Figure S2. HPLC profile, mass spectra (in positive ionization mode) and UV absorbance spectrum of indolin-2-one standard.

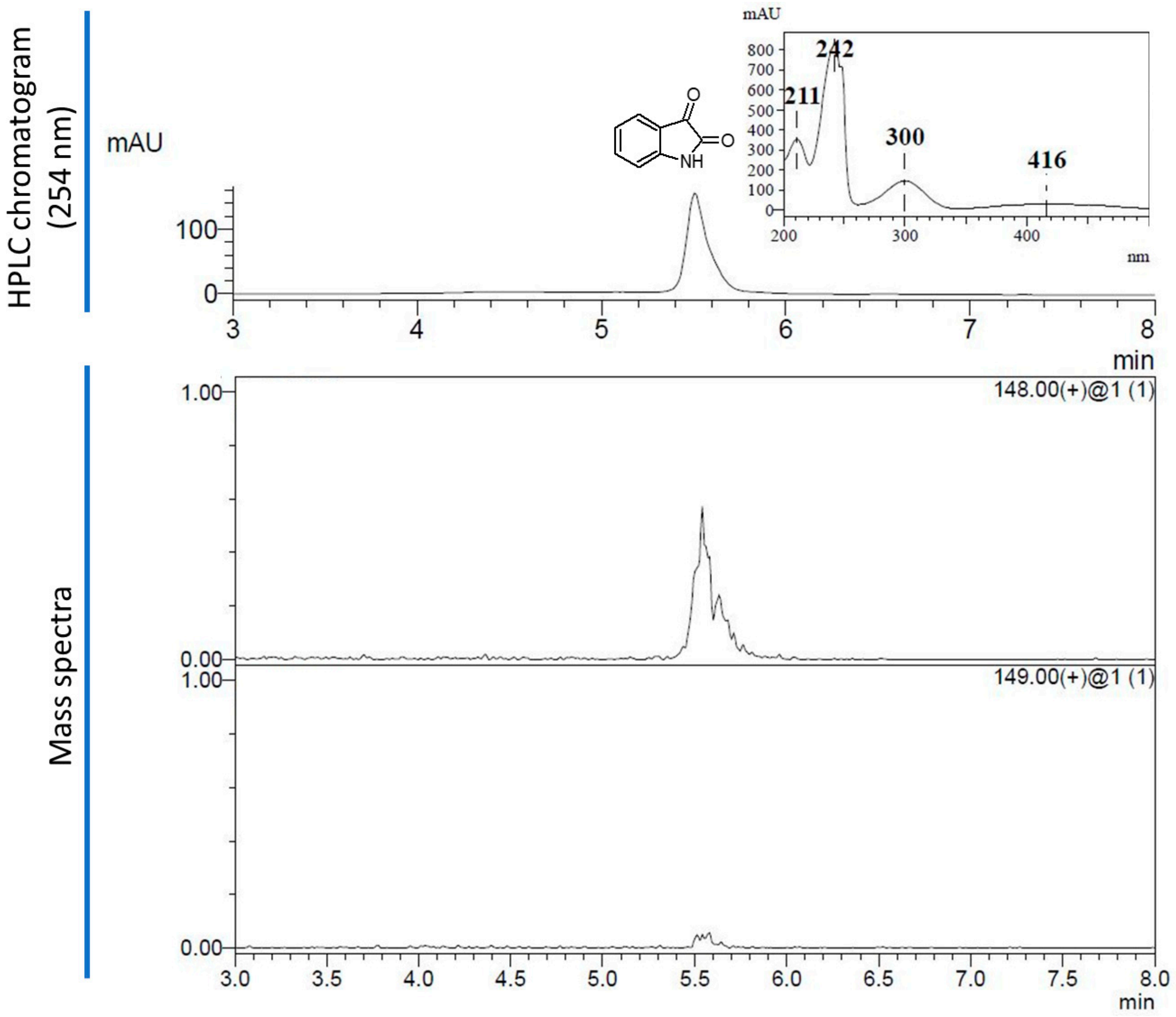

Figure S3. HPLC profile, mass spectra (in positive ionization mode) and UV absorbance spectrum of isatin standard.

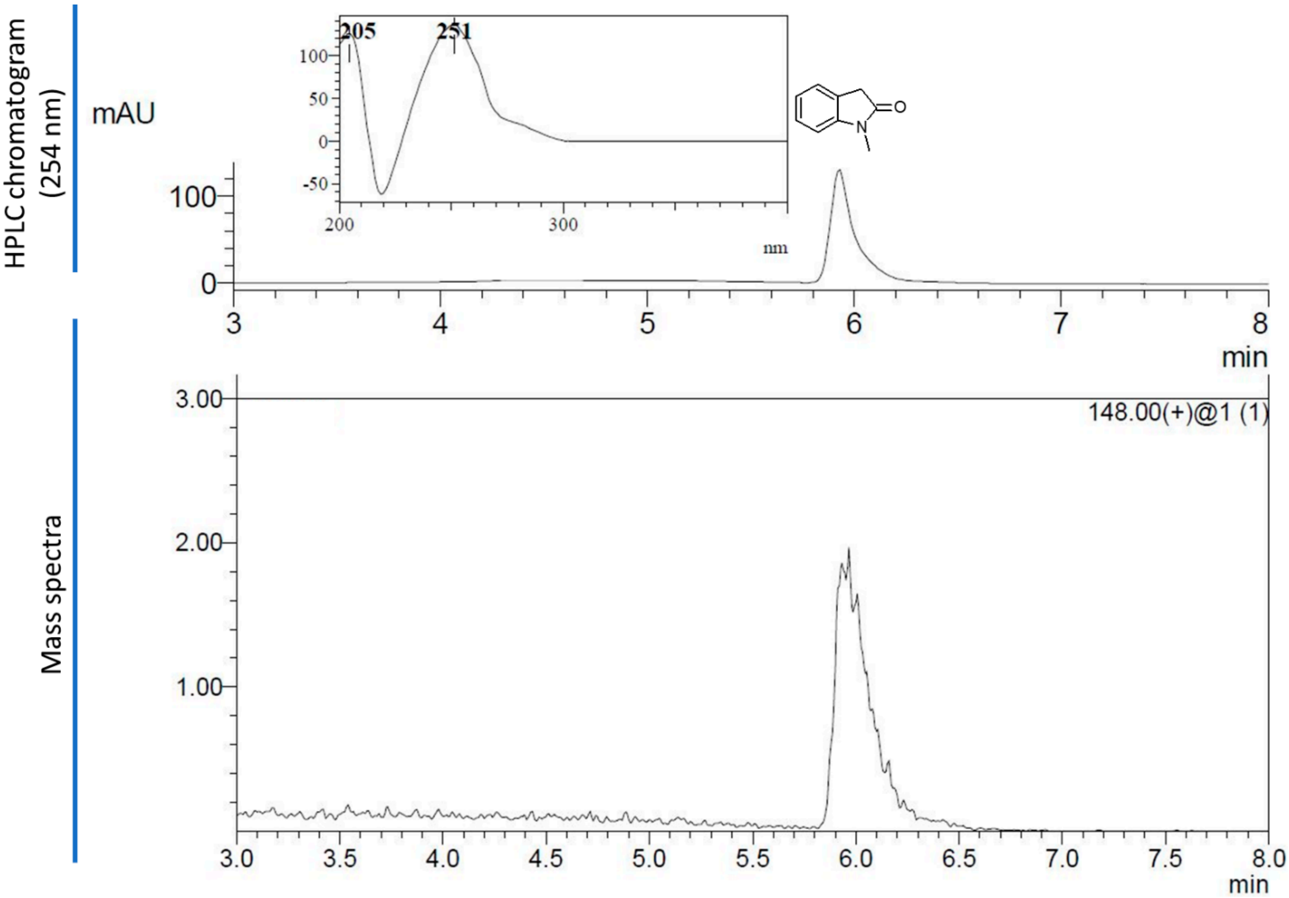

Figure S4. HPLC profile, mass spectra (in positive ionization mode) and UV absorbance spectrum of 1-methylindolin-2-one standard.

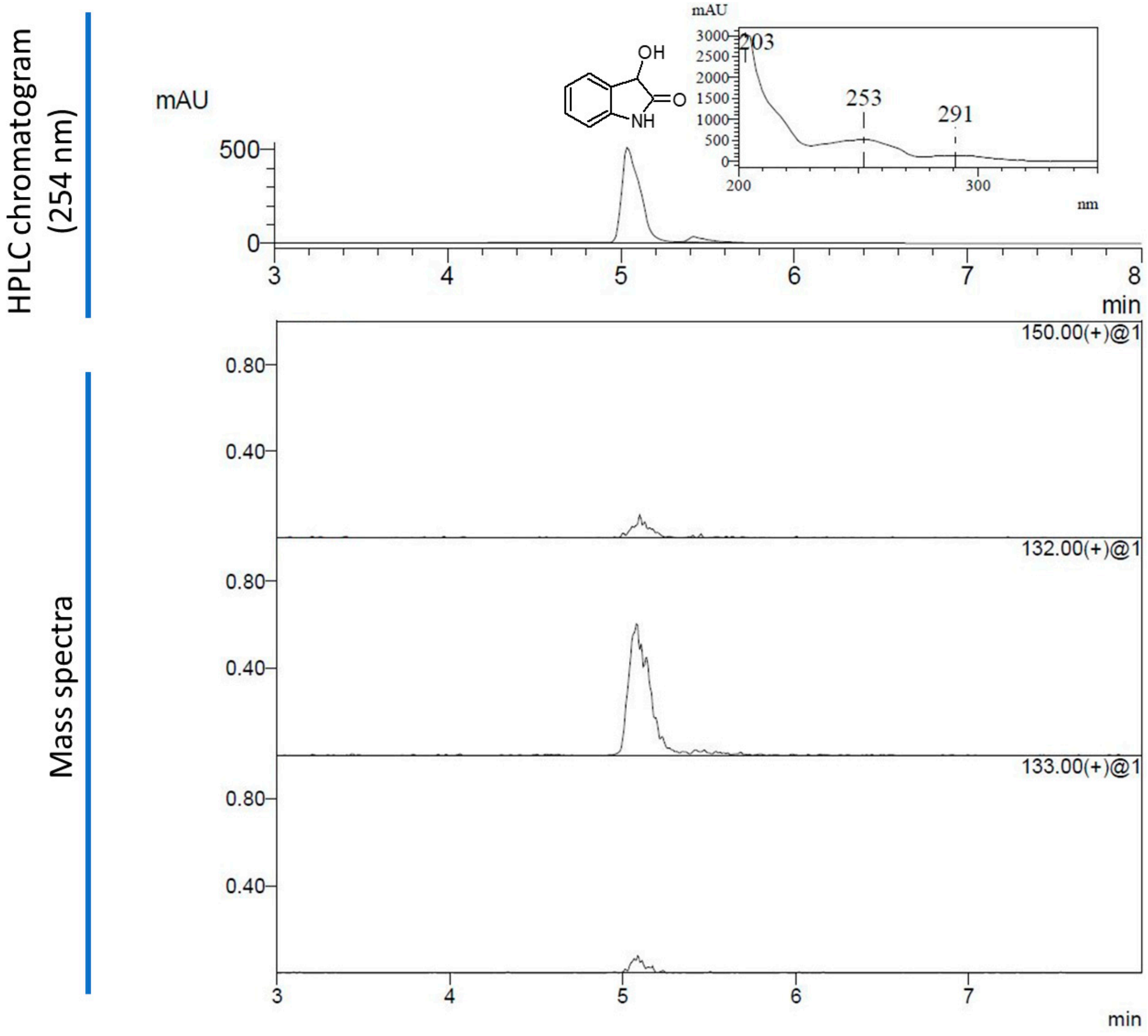

Figure S5. HPLC profile, mass spectra (in positive ionization mode) and UV absorbance spectrum of 3-hydroxyindolin-2-one standard.

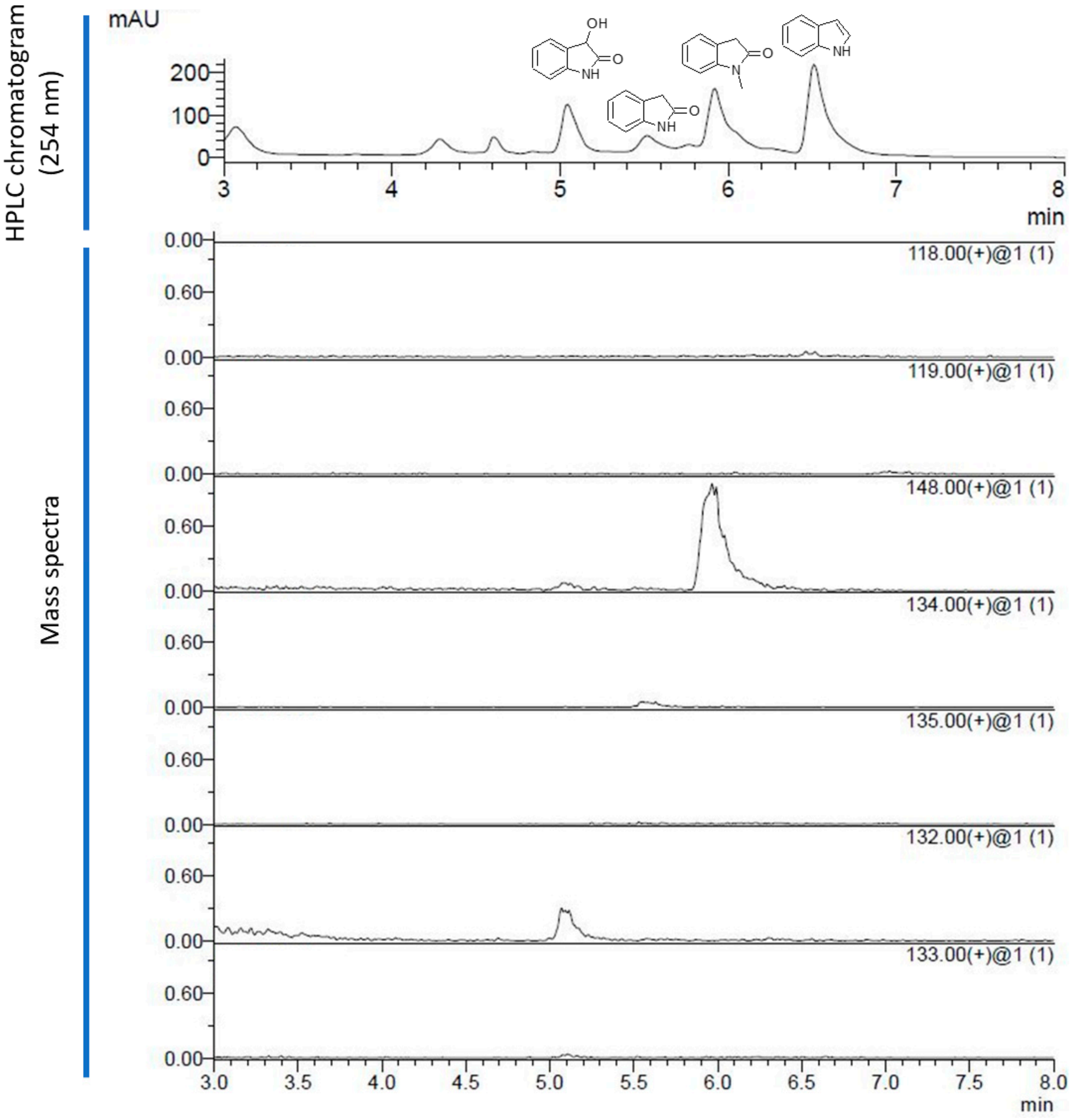

Figure S6. HPLC profile, mass spectra (in positive ionization mode) and UV absorbance spectrum of indole, indolin-2-one, isatin and 3-hydroxyindolin-2-one in small intestine.

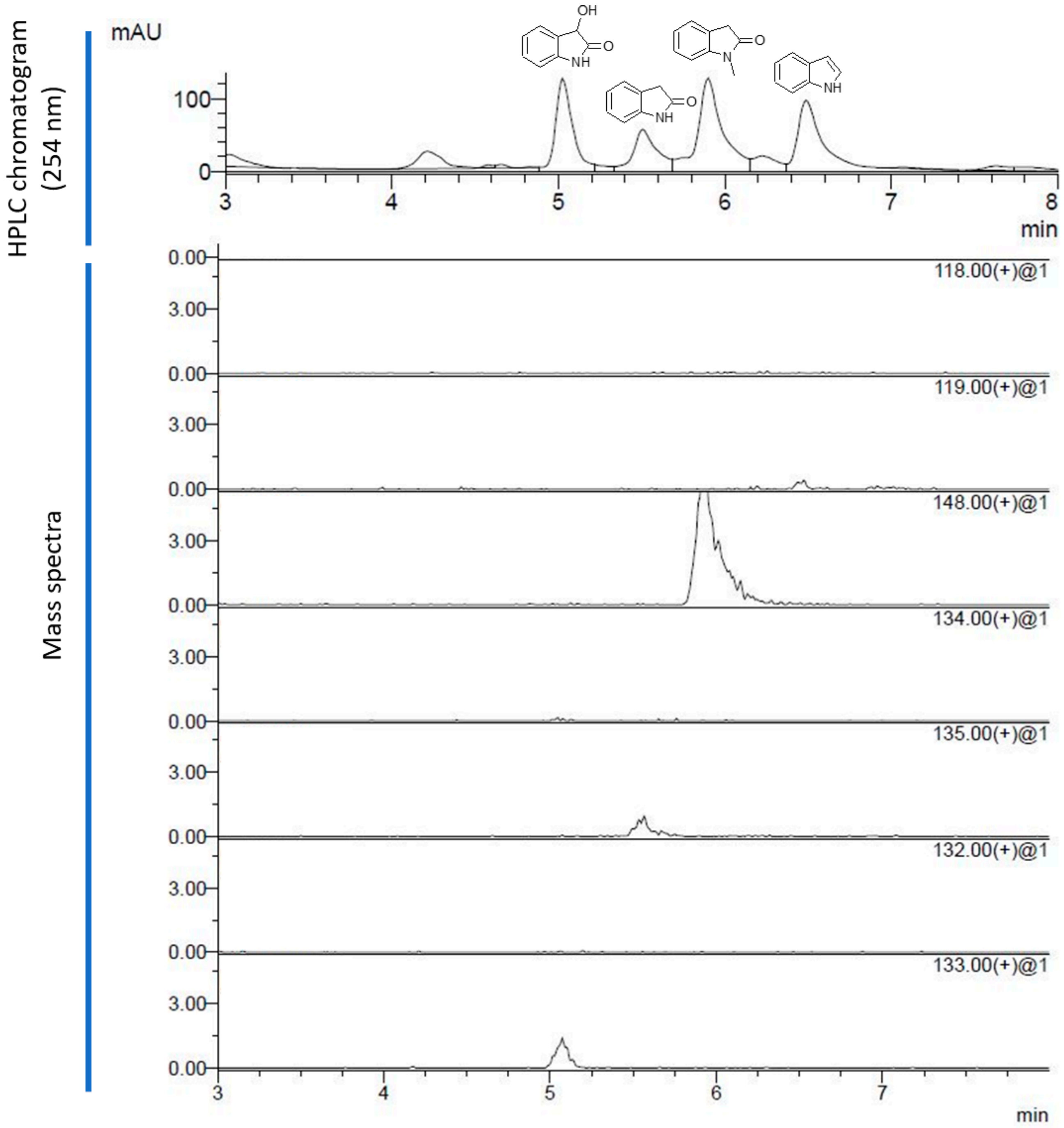

Figure S7. HPLC profile, mass spectra (in positive ionization mode) and UV absorbance spectrum of indole, indolin-2-one, isatin and 3-hydroxyindolin-2-one in small intestine after 13C isotope-carrying indole administration.

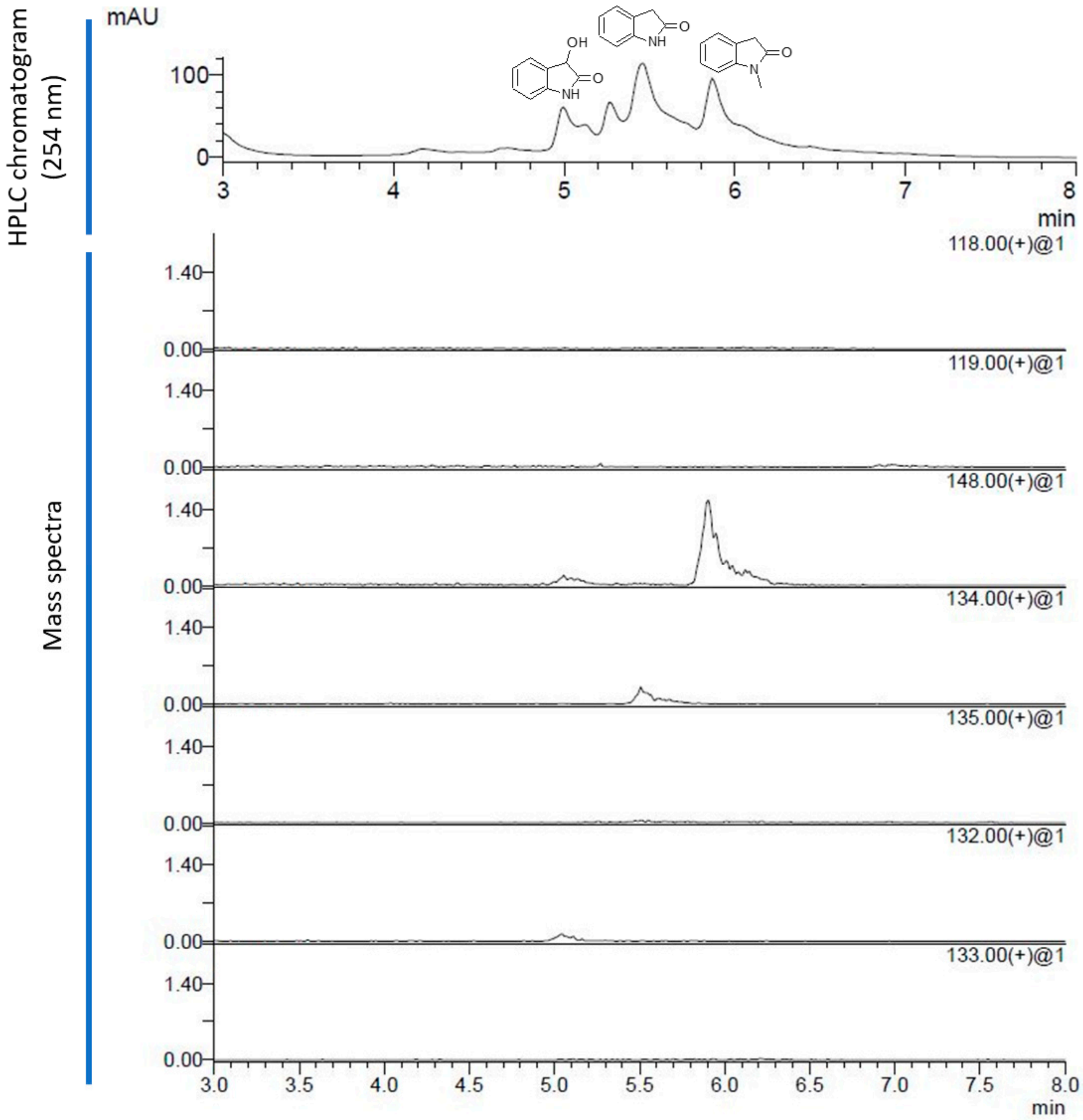

Figure S8. HPLC profile, mass spectra (in positive ionization mode) and UV absorbance spectrum of indole, indolin-2-one, isatin and 3-hydroxyindolin-2-one in cecum.

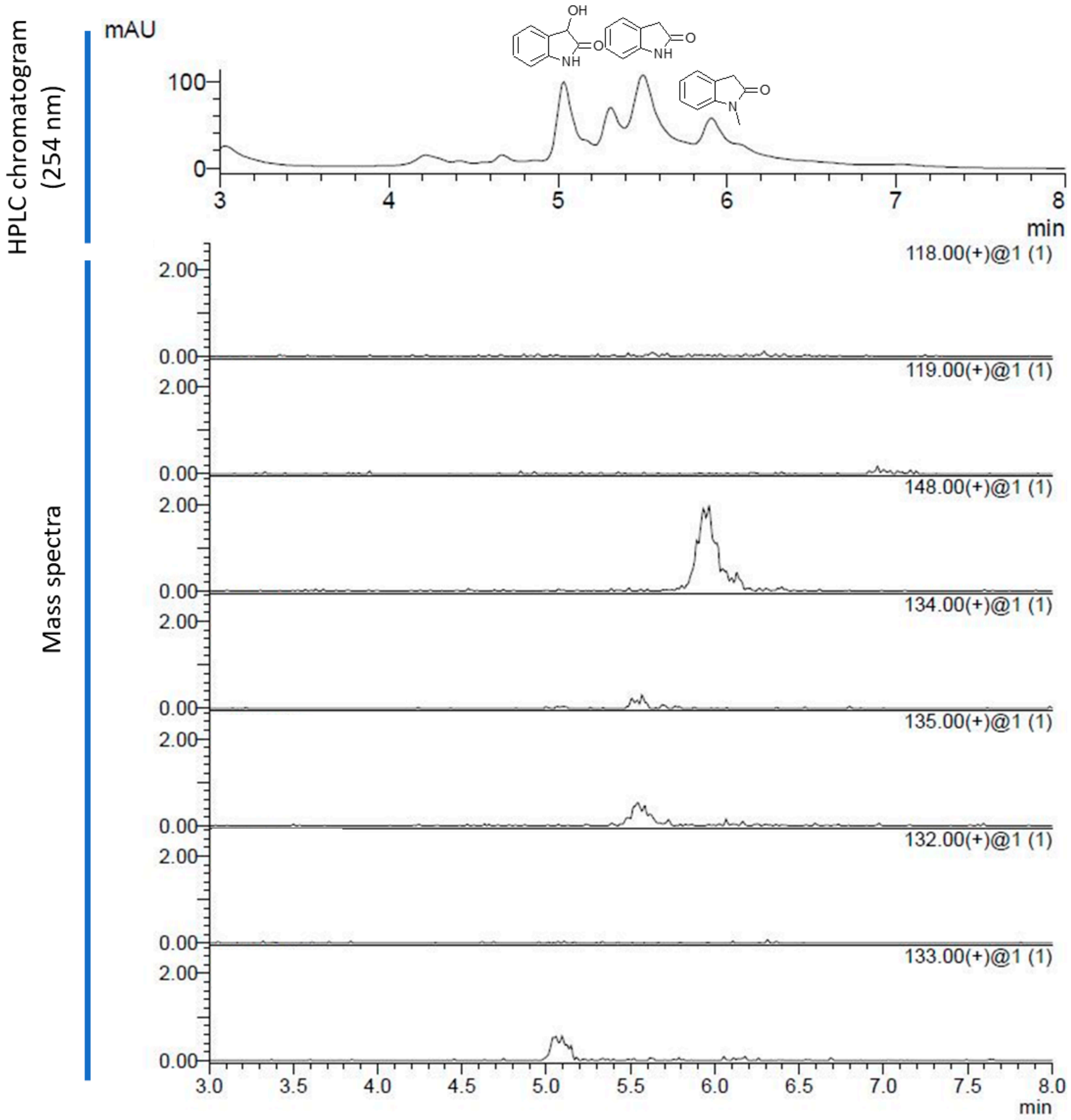

Figure S9. HPLC profile, mass spectra (in positive ionization mode) and UV absorbance spectrum of indole, indolin-2-one, isatin and 3-hydroxyindolin-2-one in cecum after 13C isotope-carrying indole administration.

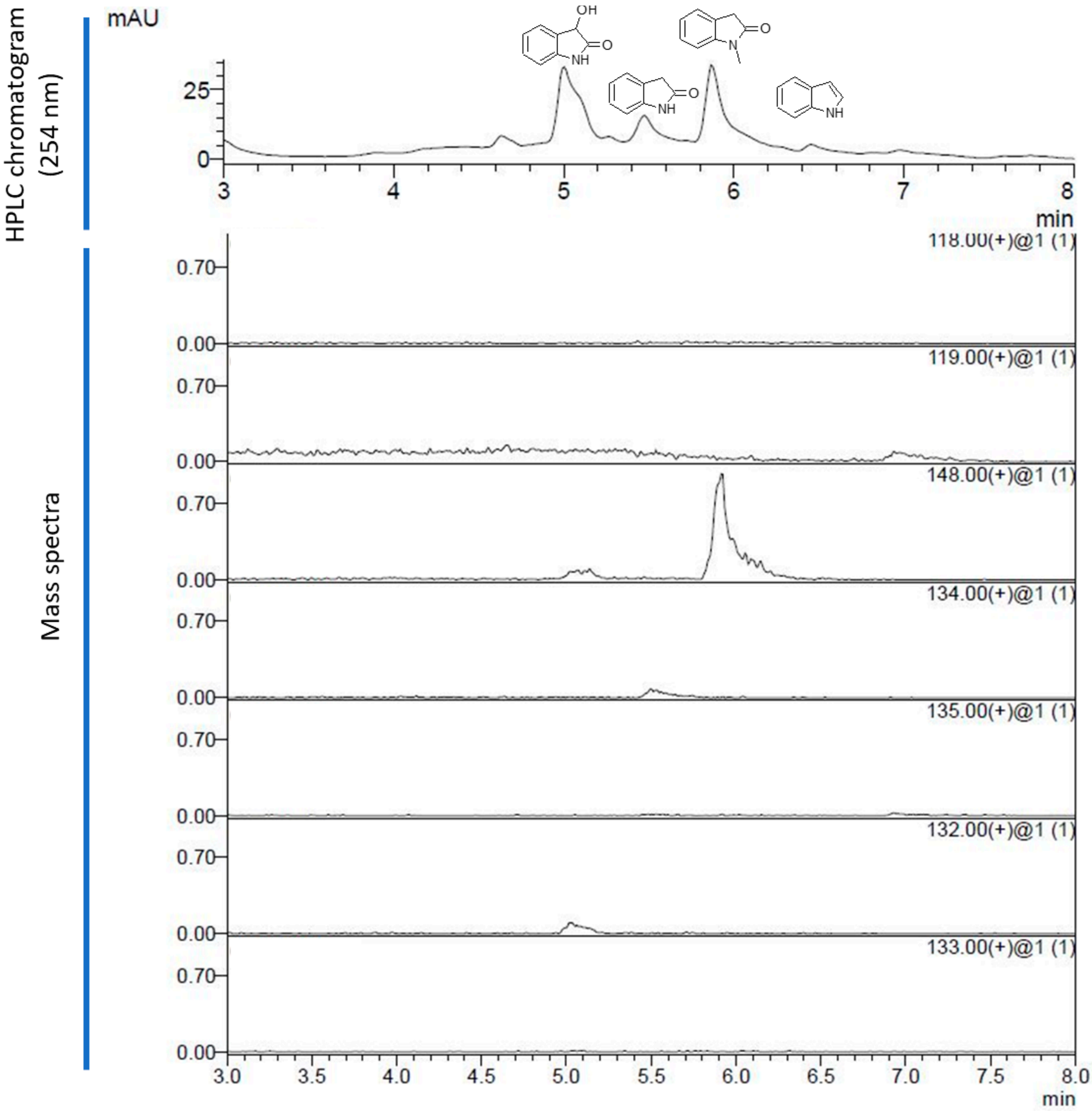

Figure S10. HPLC profile, mass spectra (in positive ionization mode) and UV absorbance spectrum of indole, indolin-2-one, isatin and 3-hydroxyindolin-2-one in large intestine.

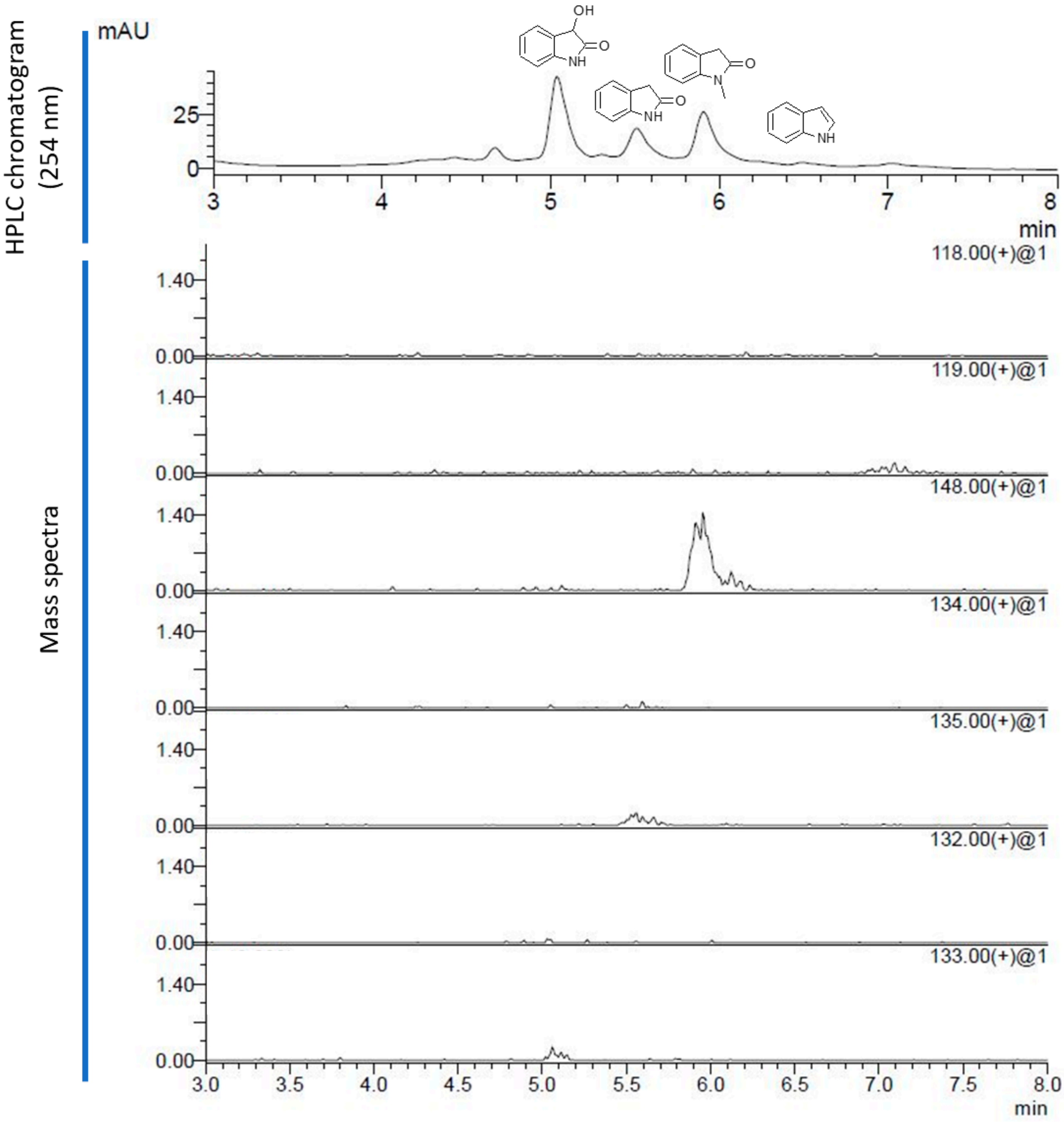

Figure S11. HPLC profile, mass spectra (in positive ionization mode) and UV absorbance spectrum of indole, indolin-2-one, isatin and 3-hydroxyindolin-2-one in large intestine after 13C isotope-carrying indole administration.

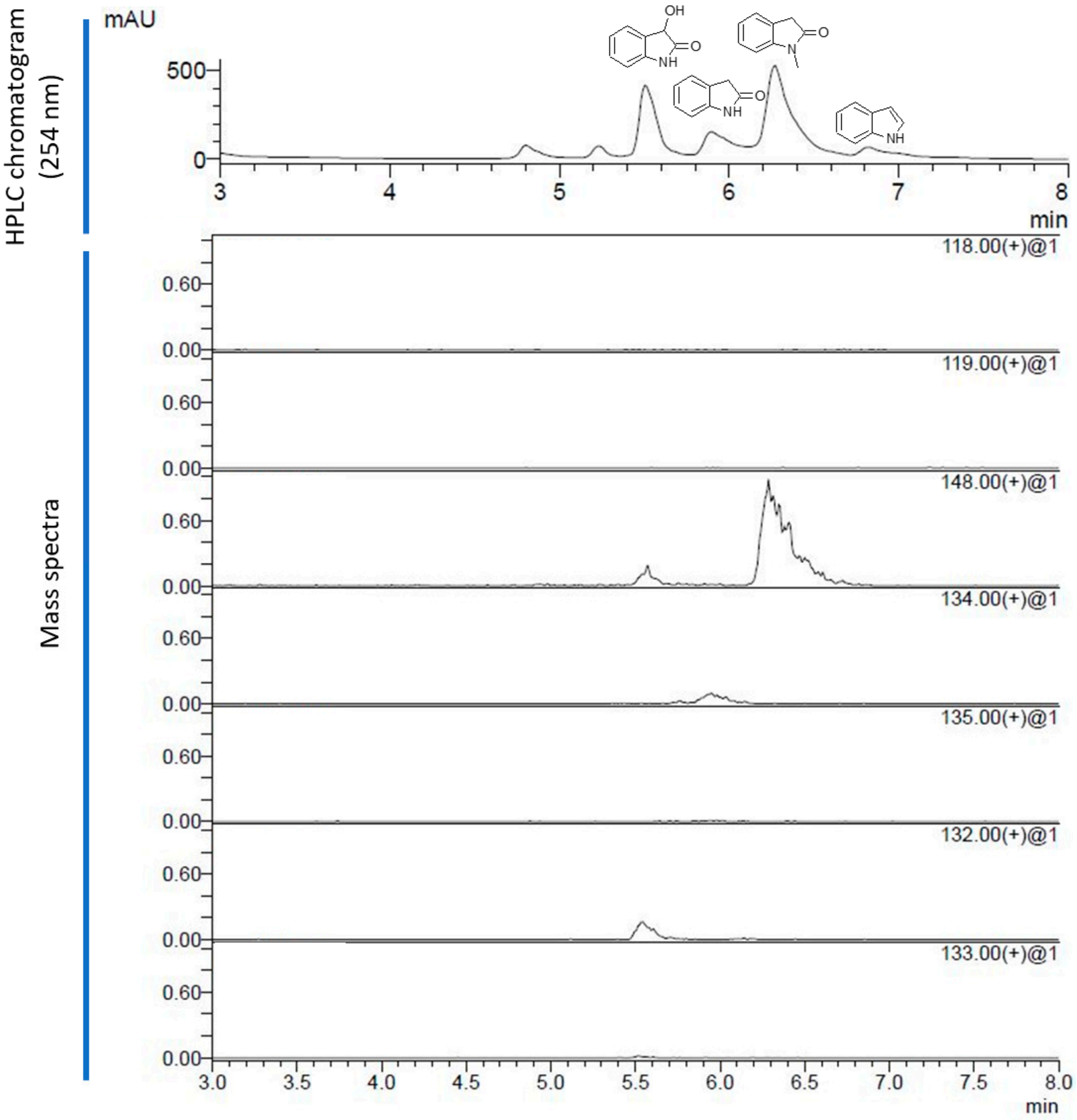

Figure S12. HPLC profile, mass spectra (in positive ionization mode) and UV absorbance spectrum of indole, indolin-2-one, isatin and 3-hydroxyindolin-2-one in liver.

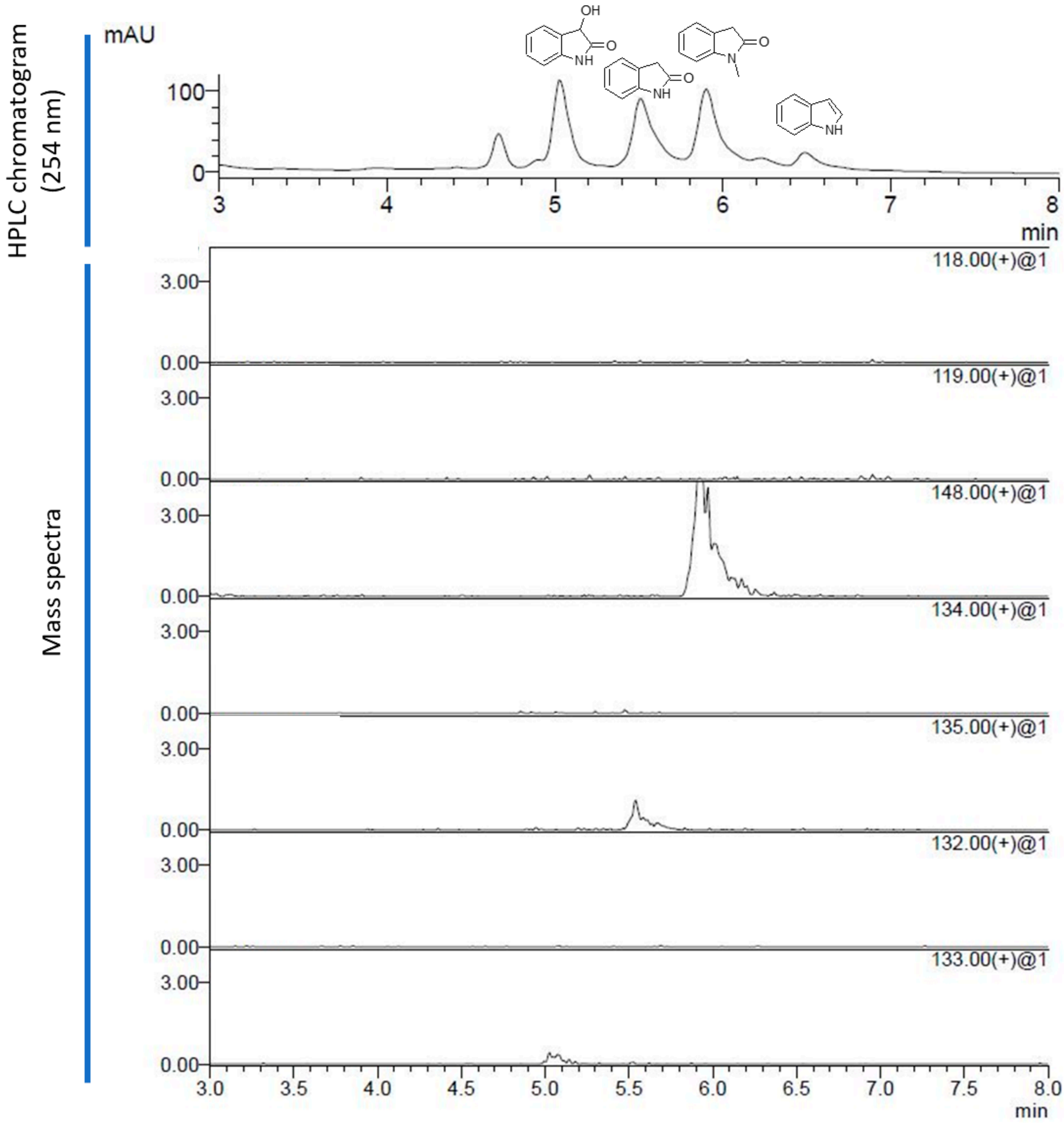

Figure S13. HPLC profile, mass spectra (in positive ionization mode) and UV absorbance spectrum of indole, indolin-2-one, isatin and 3-hydroxyindolin-2-one in liver after 13C isotope-carrying indole administration.

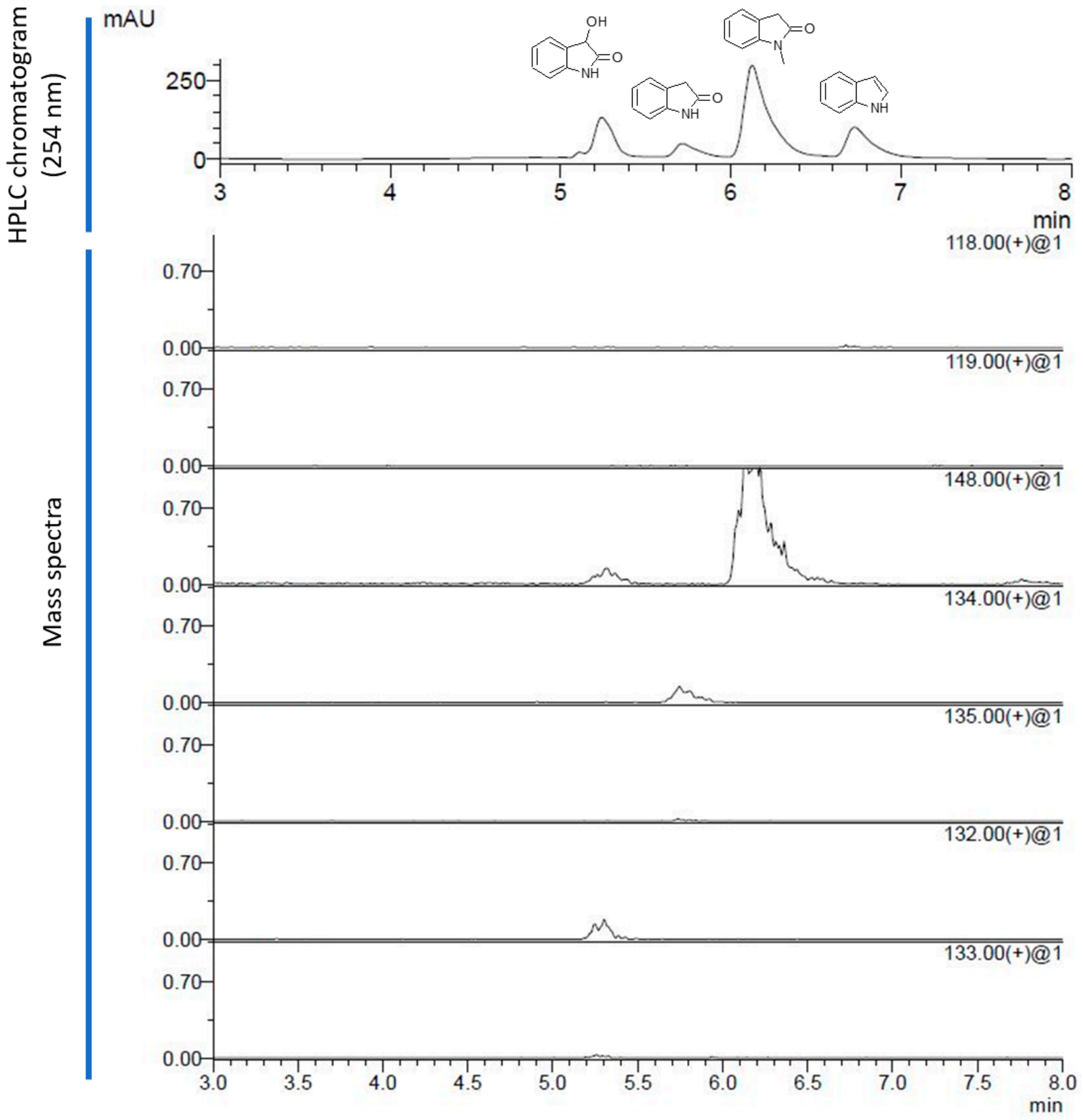

Figure S14. HPLC profile, mass spectra (in positive ionization mode) and UV absorbance spectrum of indole, indolin-2-one, isatin and 3-hydroxyindolin-2-one in brain.

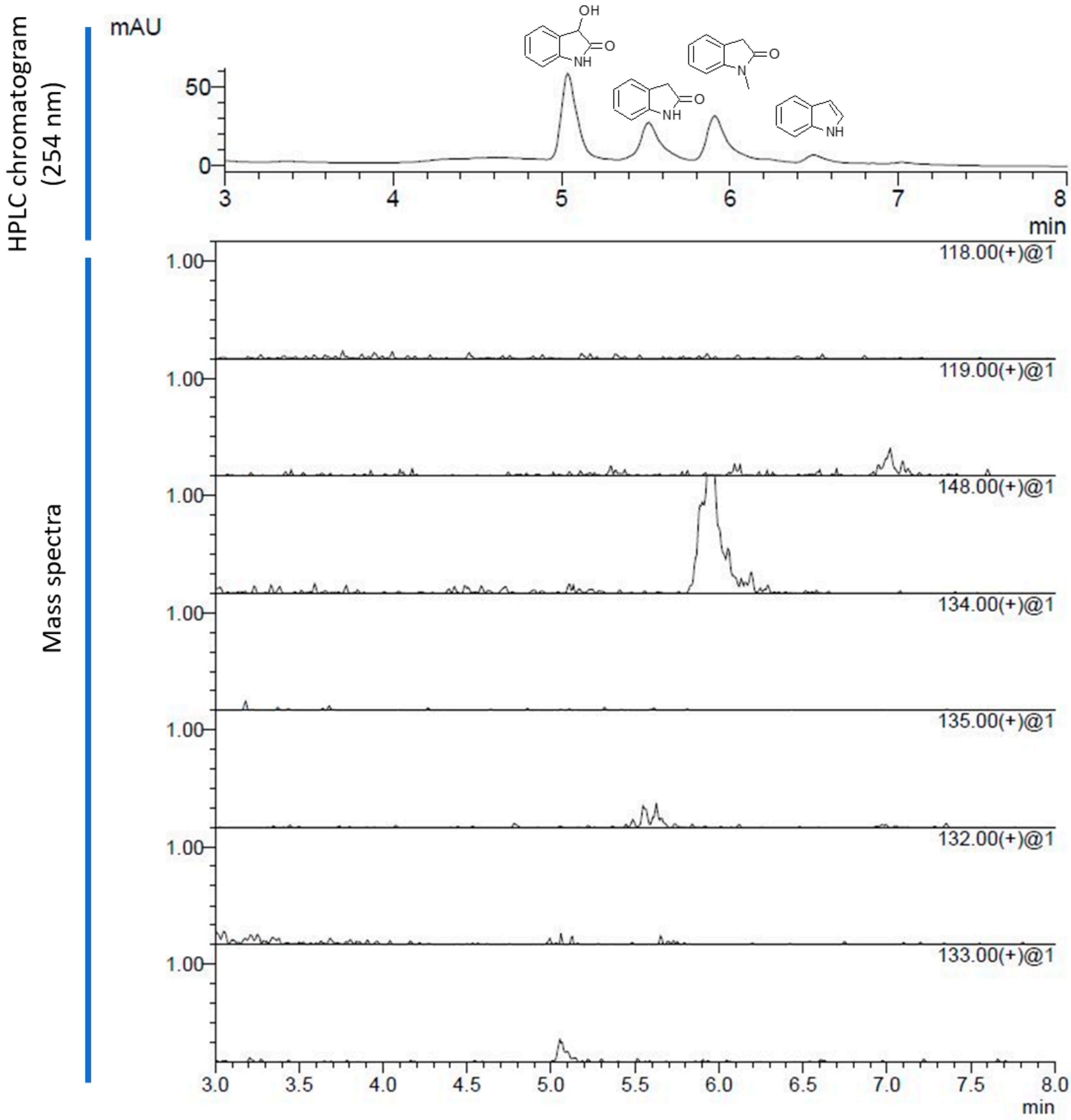

Figure S15. HPLC profile, mass spectra (in positive ionization mode) and UV absorbance spectrum of indole, indolin-2-one, isatin and 3-hydroxyindolin-2-one in brain after 13C isotope-carrying indole administration.

Table S1. Concentrations of indole metabolites in liver extracts. BDL – below detection limit.

	Sample no.	Time	Feeding	Indole, nmol/g	Indolin-2-one, nmol/g	Isatin, nmol/g	3-hydroxyindolin-2-one, nmol/g	*N*-methylindolin-2-one, mM	Recovery, %
**Liver**	1	30 min	Indole	156.1	37.26	24.42	7.43	0.035	69.2
**Liver**	2	30 min	Indole	54.56	32.76	25.25	14.12	0.035	70.1
**Liver**	3	30 min	Indole	3.21	25.74	19.84	15.72	0.034	68.0
**Liver**	4	30 min	Control	BDL	BDL	BDL	BDL	0.028	57.0
**Liver**	5	30 min	Control	BDL	BDL	BDL	BDL	0.028	55.4
**Liver**	6	30 min	Control	BDL	BDL	BDL	BDL	0.031	62.8
**Liver**	1	1 h	Indole	BDL	8.03	31.28	64.90	0.030	60.2
**Liver**	2	1 h	Indole	BDL	4.44	23.36	46.68	0.033	66.7
**Liver**	3	1 h	Indole	BDL	13.63	43.39	86.61	0.035	69.3
**Liver**	4	1 h	Control	BDL	BDL	BDL	BDL	0.031	61.3
**Liver**	5	1 h	Control	BDL	BDL	BDL	BDL	0.051	102.6
**Liver**	6	1 h	Control	BDL	BDL	BDL	BDL	0.047	94.0
**Liver**	1	2 h	Indole	67.36	BDL	BDL	-1.76	0.043	85.0
**Liver**	2	2 h	Indole	3.05	4.13	32.68	63.22	0.055	110.5
**Liver**	3	2 h	Indole	414.1	13.37	19.40	BDL	0.046	92.0
**Liver**	4	2 h	Control	BDL	BDL	BDL	BDL	0.057	114.7
**Liver**	5	2 h	Control	BDL	BDL	BDL	BDL	0.049	97.0
**Liver**	6	2 h	Control	BDL	BDL	BDL	BDL	0.047	94.8
**Liver**	1	4 h	Indole	8.76	BDL	27.29	18.61	0.037	74.4

**Liver**	2	4 h	Indole	0.00	BDL	BDL	BDL	0.034	68.6
**Liver**	3	4 h	Indole	97.7	BDL	BDL	6.72	0.028	56.8
**Liver**	4	4 h	Control	BDL	BDL	BDL	BDL	0.039	77.0
**Liver**	5	4 h	Control	BDL	BDL	BDL	BDL	0.034	67.4
**Liver**	6	4 h	Control	BDL	BDL	BDL	BDL	0.032	63.5
**Liver**	1	6 h	Indole	80.7	BDL	BDL	BDL	0.029	58.9
**Liver**	2	6 h	Indole	58.6	BDL	BDL	BDL	0.030	60.9
**Liver**	3	6 h	Indole	BDL	BDL	BDL	BDL	0.030	60.9
**Liver**	4	6 h	Control	BDL	BDL	BDL	BDL	0.033	65.5
**Liver**	5	6 h	Control	BDL	BDL	BDL	BDL	0.033	65.6
**Liver**	6	6 h	Control	BDL	BDL	BDL	BDL	0.035	69.9

Table S2. Concentrations of indole metabolites in brain extracts. BDL – below detection limit.

	Sample no.	Time	Feeding	Indole, nmol/g	Indolin-2-one, nmol/g	Isatin, nmol/g	3-hydroxyindolin-2-one, nmol/g	*N*-methylindolin-2-one, mM	Recovery, %
**Brain**	1	30 min	Indole	59.54	23.56	BDL	54.77	0.025	50.9
**Brain**	2	30 min	Indole	147.88	28.42	BDL	76.19	0.025	50.7
**Brain**	3	30 min	Indole	22.10	22.65	BDL	69.87	0.039	77.1
**Brain**	4	30 min	Control	BDL	BDL	BDL	BDL	0.030	60.2
**Brain**	5	30 min	Control	BDL	BDL	BDL	BDL	0.031	61.1
**Brain**	6	30 min	Control	BDL	BDL	BDL	BDL	0.035	69.4
**Brain**	1	1 h	Indole	4.75	15.73	BDL	65.73	0.032	63.9
**Brain**	2	1 h	Indole	BDL	6.80	BDL	37.10	0.035	69.9
**Brain**	3	1 h	Indole	5.84	14.26	BDL	68.54	0.031	62.0
**Brain**	4	1 h	Control	BDL	BDL	BDL	BDL	0.033	66.8
**Brain**	5	1 h	Control	BDL	BDL	BDL	BDL	0.033	65.3
**Brain**	6	1 h	Control	BDL	BDL	BDL	BDL	0.027	54.3
**Brain**	1	2 h	Indole	BDL	BDL	BDL	BDL	0.027	54.5
**Brain**	2	2 h	Indole	BDL	6.80	BDL	BDL	0.032	64.8
**Brain**	3	2 h	Indole	BDL	BDL	BDL	BDL	0.028	55.6
**Brain**	4	2 h	Control	BDL	BDL	BDL	BDL	0.027	53.6
**Brain**	5	2 h	Control	BDL	BDL	BDL	BDL	0.033	66.2
**Brain**	6	2 h	Control	BDL	BDL	BDL	BDL	0.030	59.7
**Brain**	1	4 h	Indole	BDL	8.33	BDL	BDL	0.030	59.4

**Brain**	2	4 h	Indole	BDL	BDL	BDL	BDL	0.030	60.8
**Brain**	3	4 h	Indole	1.41	BDL	BDL	BDL	0.017	34.3
**Brain**	4	4 h	Control	BDL	BDL	BDL	BDL	0.015	29.8
**Brain**	5	4 h	Control	BDL	BDL	BDL	BDL	0.043	86.2
**Brain**	6	4 h	Control	BDL	BDL	BDL	BDL	0.041	81.8
**Brain**	1	6 h	Indole	BDL	BDL	BDL	BDL	0.044	88.6
**Brain**	2	6 h	Indole	BDL	BDL	BDL	BDL	0.032	63.7
**Brain**	3	6 h	Indole	BDL	BDL	BDL	BDL	0.025	50.6
**Brain**	4	6 h	Control	BDL	BDL	BDL	BDL	0.033	65.5
**Brain**	5	6 h	Control	BDL	BDL	BDL	BDL	0.035	70.3
**Brain**	6	6 h	Control	BDL	BDL	BDL	BDL	0.022	44.0

Table S3. Concentrations of indole metabolites in plasma. BDL – below detection limit.

	Sample no.	Time	Feeding	Indole, nmol/g	Indolin-2-one, nmol/g	Isatin, nmol/g	3-hydroxyindolin-2-one, nmol/g	*N*-methylindolin-2-one, mM	Recovery, %
**Plasma**	1	30 min	Indole	170.99	43.38	68.50	BDL	0.041	82.8
**Plasma**	2	30 min	Indole	96.67	40.32	61.51	BDL	0.037	74.2
**Plasma**	3	30 min	Indole	15.31	18.52	24.53	BDL	0.026	52.0
**Plasma**	4	30 min	Control	0.00	0.00	0.00	BDL	0.035	70.2
**Plasma**	5	30 min	Control	0.00	0.00	0.00	BDL	0.011	22.8
**Plasma**	6	30 min	Control	0.00	0.00	0.00	BDL	0.037	74.0
**Plasma**	1	1 h	Indole	6.73	19.43	47.90	BDL	0.039	78.4
**Plasma**	2	1 h	Indole	0.00	0.00	16.62	BDL	0.043	86.7
**Plasma**	3	1 h	Indole	10.19	8.52	63.58	BDL	0.040	80.9
**Plasma**	4	1 h	Control	0.00	0.00	0.00	BDL	0.038	76.6
**Plasma**	5	1 h	Control	0.00	0.00	0.00	BDL	0.037	74.0
**Plasma**	6	1 h	Control	0.00	0.00	0.00	BDL	0.040	79.7
**Plasma**	1	2 h	Indole	0.00	0.00	0.00	BDL	0.040	79.6
**Plasma**	2	2 h	Indole	5.68	3.05	22.58	BDL	0.042	83.9
**Plasma**	3	2 h	Indole	0.00	0.00	0.00	BDL	0.024	48.6
**Plasma**	4	2 h	Control	0.00	0.00	0.00	BDL	0.050	99.4
**Plasma**	5	2 h	Control	0.00	0.00	0.00	BDL	0.048	95.8
**Plasma**	6	2 h	Control	0.00	0.00	0.00	BDL	0.041	82.8
**Plasma**	1	4 h	Indole	0.00	4.94	0.00	BDL	0.045	90.7
**Plasma**	2	4 h	Indole	0.00	7.02	0.00	BDL	0.035	69.6
**Plasma**	3	4 h	Indole	3.97	5.84	1.34	15.47	0.028	55.5
**Plasma**	4	4 h	Control	0.00	0.00	0.00	BDL	0.025	50.3
**Plasma**	5	4 h	Control	0.00	0.00	0.00	BDL	0.024	48.5
**Plasma**	6	4 h	Control	0.00	0.00	0.00	BDL	0.024	49.0
**Plasma**	1	6 h	Indole	0.00	0.00	0.00	BDL	0.037	73.9
**Plasma**	2	6 h	Indole	0.00	0.00	0.00	BDL	0.037	73.2
**Plasma**	3	6 h	Indole	0.00	0.00	0.00	BDL	0.003	6.5
**Plasma**	4	6 h	Control	0.00	0.00	0.00	BDL	0.026	52.4
**Plasma**	5	6 h	Control	0.00	0.00	0.00	BDL	0.033	66.2
**Plasma**	6	6 h	Control	0.00	0.00	0.00	BDL	0.029	58.4

Table S4. Concentrations of indole metabolites in small intestine (SI) extracts. BDL – below detection limit.

	Sample no.	Time	Feeding	Indole, nmol/g	Indolin-2-one, nmol/g	Isatin, nmol/g	3-hydroxyindolin-2-one, nmol/g	*N*-methylindolin-2-one, mM	Recovery, %
SI	1	30 min	Indole	138.09	BDL	21.27	76.51	0.027	53.5
SI	2	30 min	Indole	1083.23	BDL	25.02	53.50	0.028	56.8
SI	3	30 min	Indole	117.59	BDL	22.05	66.77	0.030	60.7
SI	4	30 min	Control	0.00	BDL	BDL	BDL	0.027	53.4
SI	5	30 min	Control	2.24	BDL	BDL	BDL	0.024	47.8
SI	6	30 min	Control	1.43	BDL	BDL	BDL	0.020	40.3
SI	1	1 h	Indole	243.28	BDL	18.66	52.78	0.039	77.5
SI	2	1 h	Indole	18.40	BDL	BDL	4.63	0.004	7.4
SI	3	1 h	Indole	76.92	BDL	88.62	BDL	0.032	64.4
SI	4	1 h	Control	1.23	BDL	BDL	BDL	0.048	95.7
SI	5	1 h	Control	2.37	BDL	BDL	BDL	0.021	41.8
SI	6	1 h	Control	2.07	BDL	BDL	BDL	0.037	73.4
SI	1	2 h	Indole	1276.35	BDL	21.97	BDL	0.025	49.3
SI	2	2 h	Indole	6.97	BDL	18.46	48.78	0.028	55.5
SI	3	2 h	Indole	3.09	BDL	4.29	BDL	0.039	78.0
SI	4	2 h	Control	3.79	BDL	BDL	BDL	0.043	86.8
SI	5	2 h	Control	0.00	BDL	BDL	BDL	0.033	65.4
SI	6	2 h	Control	0.00	BDL	BDL	BDL	0.036	72.5

SI	1	4 h	Indole	30.43	BDL	BDL	BDL	0.025	50.7
SI	2	4 h	Indole	3.32	BDL	BDL	24.91	0.040	80.8
SI	3	4 h	Indole	284.77	BDL	17.29	BDL	0.032	64.0
SI	4	4 h	Control	0.00	BDL	BDL	BDL	0.028	56.1
SI	5	4 h	Control	1.86	BDL	BDL	BDL	0.029	57.8
SI	6	4 h	Control	1.55	BDL	BDL	BDL	0.025	49.5
SI	1	6 h	Indole	110.07	BDL	85.15	BDL	0.045	89.4
SI	2	6 h	Indole	110.77	BDL	59.01	BDL	0.043	85.6
SI	3	6 h	Indole	3.78	BDL	31.08	BDL	0.044	88.8
SI	4	6 h	Control	0.00	BDL	BDL	BDL	0.032	64.2
SI	5	6 h	Control	1.77	BDL	BDL	BDL	0.034	67.7
SI	6	6 h	Control	0.00	BDL	BDL	BDL	0.040	80.5

Table S5. Concentrations of indole metabolites in cecum extracts. BDL – below detection limit.

	Sample no.	Time	Feeding	Indole, nmol/g	Indolin-2-one, nmol/g	Isatin, nmol/g	3-hydroxyindolin-2-one, nmol/g	*N*-methylindolin-2-one, mM	Recovery, %
**Cecum**	1	30 min	Indole	37.54	BDL	BDL	22.14	0.024	48.3
**Cecum**	2	30 min	Indole	22.73	BDL	BDL	29.66	0.035	70.2
**Cecum**	4	30 min	Control	9.94	BDL	BDL	BDL	0.027	53.5
**Cecum**	5	30 min	Control	14.89	BDL	BDL	BDL	0.022	43.0
**Cecum**	6	30 min	Control	21.09	BDL	BDL	BDL	0.019	38.1
**Cecum**	1	1 h	Indole	29.05	BDL	BDL	18.19	0.031	61.2
**Cecum**	2	1 h	Indole	32.96	BDL	BDL	12.89	0.030	60.3
**Cecum**	3	1 h	Indole	48.99	BDL	BDL	17.05	0.031	62.6
**Cecum**	4	1 h	Control	27.69	BDL	BDL	BDL	0.027	53.8
**Cecum**	5	1 h	Control	31.82	BDL	BDL	BDL	0.033	65.7
**Cecum**	6	1 h	Control	37.34	BDL	BDL	BDL	0.025	50.7
**Cecum**	1	2 h	Indole	7.99	BDL	BDL	7.06	0.030	60.6
**Cecum**	2	2 h	Indole	0.00	BDL	BDL	19.80	0.037	74.3
**Cecum**	3	2 h	Indole	24.86	BDL	BDL	10.80	0.030	59.9
**Cecum**	4	2 h	Control	23.66	BDL	BDL	BDL	0.025	50.6
**Cecum**	5	2 h	Control	0.00	BDL	BDL	BDL	0.031	62.9
**Cecum**	6	2 h	Control	0.00	BDL	BDL	BDL	0.027	53.3
**Cecum**	1	4 h	Indole	0.00	BDL	BDL	17.61	0.031	62.6
**Cecum**	2	4 h	Indole	0.00	BDL	BDL	21.81	0.031	62.9

**Cecum**	3	4 h	Indole	12.47	BDL	BDL	BDL	0.040	80.8
**Cecum**	4	4 h	Control	7.69	BDL	BDL	BDL	0.029	57.0
**Cecum**	5	4 h	Control	0.00	BDL	BDL	BDL	0.044	87.3
**Cecum**	6	4 h	Control	18.75	BDL	BDL	BDL	0.035	70.3
**Cecum**	1	6 h	Indole	0.00	BDL	BDL	35.22	0.035	70.5
**Cecum**	2	6 h	Indole	0.00	BDL	BDL	BDL	0.025	50.2
**Cecum**	3	6 h	Indole	0.00	BDL	BDL	13.51	0.041	81.9
**Cecum**	4	6 h	Control	34.53	BDL	BDL	BDL	0.034	68.5
**Cecum**	5	6 h	Control	11.51	BDL	BDL	BDL	0.029	58.7
**Cecum**	6	6 h	Control	20.80	BDL	BDL	BDL	0.032	64.5

Table S6. Concentrations of indole metabolites in large intestine (LI) extracts. BDL – below detection limit.

	Sample no.	Time	Feeding	Indole, nmol/g	Indolin-2-one, nmol/g	Isatin, nmol/g	3-hydroxyindolin-2-one, nmol/g	*N*-methylindolin-2-one, mM	Recovery, %
LI	1	30 min	Indole	119.49	BDL	5.12	45.32	0.032	64.3
LI	2	30 min	Indole	137.49	BDL	BDL	75.39	0.036	71.0
LI	3	30 min	Indole	66.42	BDL	BDL	41.71	0.033	65.7
LI	4	30 min	Control	8.80	BDL	BDL	BDL	0.035	70.6
LI	5	30 min	Control	5.57	BDL	BDL	BDL	0.034	67.1
LI	6	30 min	Control	2.63	BDL	BDL	BDL	0.040	80.6
LI	1	1 h	Indole	4.64	BDL	1.34	30.45	0.032	63.9
LI	2	1 h	Indole	2.89	BDL	4.73	6.37	0.030	60.1
LI	3	1 h	Indole	70.74	BDL	27.77	58.67	0.049	97.9
LI	4	1 h	Control	5.01	BDL	BDL	BDL	0.047	93.4
LI	5	1 h	Control	BDL	BDL	BDL	BDL	0.034	67.5
LI	6	1 h	Control	13.85	BDL	BDL	BDL	0.038	75.4
LI	1	2 h	Indole	2.89	BDL	BDL	BDL	0.026	52.7
LI	2	2 h	Indole	7.18	BDL	6.65	BDL	0.027	54.7
LI	3	2 h	Indole	278.22	BDL	BDL	BDL	0.048	95.1
LI	4	2 h	Control	2.76	BDL	BDL	BDL	0.049	98.7
LI	5	2 h	Control	BDL	BDL	BDL	BDL	0.049	98.2
LI	6	2 h	Control	7.49	BDL	BDL	BDL	0.044	88.3

**LI**	1	4 h	Indole	30.39	BDL	BDL	BDL	0.048	96.1
**LI**	2	4 h	Indole	11.51	BDL	BDL	BDL	0.036	72.7
**LI**	3	4 h	Indole	34.93	BDL	BDL	BDL	0.043	86.0
**LI**	4	4 h	Control	6.83	BDL	BDL	BDL	0.040	79.7
**LI**	5	4 h	Control	4.42	BDL	BDL	BDL	0.026	52.1
**LI**	6	4 h	Control	BDL	BDL	BDL	BDL	0.038	76.2
**LI**	1	6 h	Indole	171.66	BDL	26.96	18.68	0.036	72.6
**LI**	2	6 h	Indole	231.01	BDL	BDL	BDL	0.038	75.6
**LI**	3	6 h	Indole	24.29	BDL	94.69	33.36	0.046	91.2
**LI**	4	6 h	Control	12.31	BDL	BDL	BDL	0.047	93.7
**LI**	5	6 h	Control	BDL	BDL	BDL	BDL	0.005	9.7
**LI**	6	6 h	Control	11.65	BDL	BDL	BDL	0.043	86.6
